# Directed evolution unlocks oxygen reactivity for a nicotine-degrading flavoenzyme

**DOI:** 10.1038/s41589-023-01426-y

**Published:** 2023-09-28

**Authors:** Mark Dulchavsky, Rishav Mitra, Kevin Wu, Joshua Li, Karli Boer, Xiaomeng Liu, Zhiyao Zhang, Cristian Vasquez, Christopher T. Clark, Kaitrin Funckes, Kokila Shankar, Selene Bonnet-Zahedi, Mohammad Siddiq, Yadira Sepulveda, Raymond T. Suhandynata, Jeremiah D. Momper, Antonio N. Calabrese, Olivier George, Frederick Stull, James C. A. Bardwell

**Affiliations:** 1 Howard Hughes Medical Institute and Department of Molecular, Cellular and Developmental Biology, University of Michigan, Ann Arbor, MI USA; 2 Cellular and Molecular Biology Program, University of Michigan, Ann Arbor, MI USA; 3 Department of Chemistry, Western Michigan University, Kalamazoo, MI USA; 4 Department of Psychiatry, University of California, San Diego, La Jolla, CA USA; 5 Department of Ecology and Evolutionary Biology, University of Michigan, Ann Arbor, MI USA; 6 School of Pharmacy and Pharmaceutical Science, University of California, San Diego, La Jolla, CA USA; 7 Department of Pathology, University of California, San Diego, La Jolla, CA USA; 8 Astbury Centre for Structural Molecular Biology, S chool of Molecular and Cellular Biology, Faculty of Biological Sciences, University of Leeds, Leeds, UK

**Keywords:** Enzyme mechanisms, Biologics, Biophysics, Structural biology, Biocatalysis

## Abstract

The flavoenzyme nicotine oxidoreductase (NicA2) is a promising injectable treatment to aid in the cessation of smoking, a behavior responsible for one in ten deaths worldwide. NicA2 acts by degrading nicotine in the bloodstream before it reaches the brain. Clinical use of NicA2 is limited by its poor catalytic activity in the absence of its natural electron acceptor CycN. Without CycN, NicA2 is instead oxidized slowly by dioxygen (O_2_), necessitating unfeasibly large doses in a therapeutic setting. Here, we report a genetic selection strategy that directly links CycN-independent activity of NicA2 to growth of *Pseudomonas putida* S16. This selection enabled us to evolve NicA2 variants with substantial improvement in their rate of oxidation by O_2_. The encoded mutations cluster around a putative O_2_ tunnel, increasing flexibility and accessibility to O_2_ in this region. These mutations further confer desirable clinical properties. A variant form of NicA2 is tenfold more effective than the wild type at degrading nicotine in the bloodstream of rats.

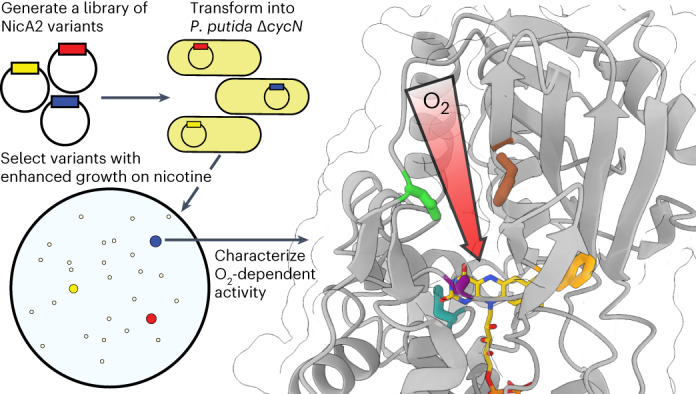

## Abstract (web summary)

The flavoenzyme nicotine oxidoreductase degrades nicotine in the bloodstream. Now, genetic selection in bacteria has been used to improve the catalytic performance of nicotine oxidoreductase, isolating variants with increased O_2_ reactivity that were more effective at degrading nicotine in the blood of rats.

## Main

The addictive potential of nicotine makes smoking cessation difficult^1^. However, when abstinence from tobacco is achieved, the personal and public health benefits are unparalleled^2^. Nicotine oxidoreductase (NicA2) is a flavin-dependent enzyme that catalyzes the degradation of nicotine into the non-psychoactive metabolite *N*-methylmyosmine (NMM)^3^. NicA2 has thus garnered interest as an injectable therapeutic to aid in the cessation of smoking. Several rodent studies have demonstrated the efficacy of NicA2, but the staggeringly large doses of the enzyme necessary to achieve therapeutic benefit (10–70 mg kg^−1^) remain a major caveat^4–7^. The reason such sizable amounts of NicA2 are required is the poor oxygen reactivity of this enzyme^8,9^.

NicA2 catalyzes reduction and oxidation reactions by first accepting electrons from nicotine^10^. This leads to the conversion of its bound flavin adenine dinucleotide (FAD) to the reduced hydroquinone form (FADH_2_). FADH_2_ must be oxidized after this reductive step to complete the catalytic cycle. Dioxygen (O_2_) is the canonical electron acceptor for the enzyme family containing NicA2, the flavin-dependent amine oxidases^11^. If O_2_ is the only substrate available to oxidize the flavin of NicA2, as is likely the case in the bloodstream, the turnover of NicA2 is extremely slow with a *k*_cat_ of ~0.007 s^−1^ (ref. ^12^). In its native organism *Pseudomonas putida* S16, NicA2 achieves a rate of catalysis four orders of magnitude faster than in vitro. The higher in vivo activity of NicA2 is due to the presence of a cytochrome *c*, termed CycN, that acts as an alternative electron acceptor to O_2_^9^. CycN is critical for the ability of this microorganism to grow using nicotine as a carbon source. However, supplementing CycN is unlikely to be useful therapeutically due to the impracticality of reconstituting electron transfer machinery in a treatment setting. A more straightforward solution to make NicA2 a suitable therapeutic is to increase its rate of reaction with the most readily available oxidant in the bloodstream, O_2_.

Due to the higher redox potential of the O_2_/H_2_O_2_ couple than that of flavins, all reduced flavins are thermodynamically poised to be oxidized by O_2_ in an essentially irreversible reaction^13^. For flavin-containing oxidases, O_2_ is a ready reactant, with rate constants of oxidation typically falling between 10^3^ and 10^6^ M^−1^ s^−1^ (ref. ^14^). By contrast, flavin-containing dehydrogenases do not efficiently facilitate a reaction between their bound flavins and O_2_, with rate constants for oxidation that can approach zero^15^. Despite roughly 100 years of study^16^, the molecular features that govern O_2_ reactivity remain obscure^14,17^. NicA2 provides an appealing opportunity to investigate how flavoenzymes can control their reactivity with O_2_ because it can be compared to structurally similar enzymes that, unlike NicA2, are reactive with O_2_^8,9,11,14^.

Because evolutionarily related flavin-containing amine oxidases readily use O_2_, we hypothesized that it should be possible to evolve NicA2 to favor O_2_ as a substrate. Others have attempted to increase the oxidation rate of NicA2 by making rational substitutions guided by the currently limited understanding of oxygen control in flavoenzymes. These have resulted in only modest improvements in the nicotine degradation rate of NicA2 (refs. ^7,8,18^). We have observed that a null mutant of NicA2’s in vivo electron acceptor CycN has a severe growth defect when *P. putida* S16 is grown in minimal medium containing nicotine as the sole carbon source. This phenotype offers us a unique opportunity to isolate NicA2 variants that can effectively use O_2_ as an electron acceptor. We isolated variants from this selection with up to an ~189-fold improvement in their rate of nicotine degradation under ambient conditions and a 10-fold increase in *k*_cat_/*K*_m_. Our results indicate that this catalytic improvement can be at least partly explained by changes in the conformational dynamics of a putative O_2_-accessible tunnel in NicA2.

Although therapeutic proteins have gained widespread clinical application, challenges including their high cost and adverse effects associated with immunogenicity remain. The utility of therapeutic proteins for preventative treatment of chronic conditions such as smoking cessation has yet to be established. Regardless, these improved NicA2 variants require a dose that is ten times lower than the wild-type enzyme to deplete nicotine in the blood of rats, making them better candidates for future preclinical studies.

## Results

### *P. putida* S16 Δ*cycN* as a selection for O_2_-reactive variants

**Fig. 1:**
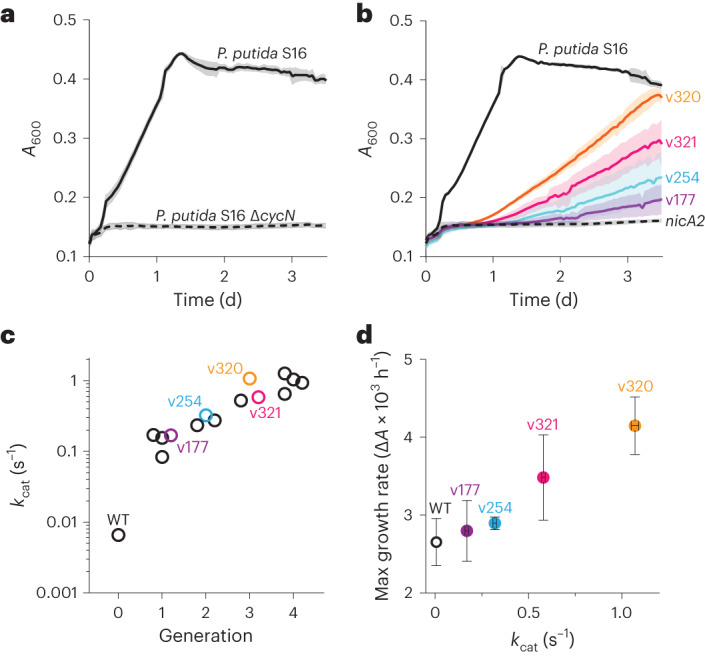
*P. putida* S16 Δ*cycN* provides a platform for genetic selection. **a**, Growth of wild-type *P. putida* S16 and Δ*cycN* in liquid culture with nicotine as the sole carbon source. The mean is plotted with error bands that represent the s.d. of three biological replicates; *A*_600_, absorbance at 600 nm. **b**, Growth of wild-type *P. putida* S16 in liquid culture with 3 mM nicotine as a sole carbon source is shown in solid black. Growth of *P. putida* S16 Δ*cycN* strains transformed with the wild-type sequence of *nicA2* in the same medium is shown by the dashed black, and the growth of *nicA2* variants coming from the selection is shown in the colored curves with their allele numbers indicated. Note that the color scheme of the variants carries over between the growth curves and the data in **c** and **d**. The mean is plotted with error bands that represent the s.d. of three biological replicates. **c**, *nicA2* variants isolated from different generations of the selection were purified and characterized for their encoded mutations and steady-state kinetic parameters (Supplementary Table 1 and Extended Data Fig. 1a). The steady-state kinetic assays in this study were performed under ambient conditions, meaning that the *k*_cat_ values discussed here actually represent an apparent *k*_cat_ for nicotine turnover at an oxygen concentration of roughly 250 µM. Note that the *y* axis has a logarithmic scale; WT, wild type. **d**, The maximum growth rate of variants transformed in *P. putida* S16 Δ*cycN* tested in **b** plotted against the determined *k*_cat_ of each variant. The mean is plotted with error bars that represent the 95% confidence intervals of three replicates for each value; Δ*A*, change in absorbance. Source data

*P. putida* S16 exploits the pyrrolidine catabolic pathway to grow using nicotine as a sole carbon source^19^. The electrons liberated by NicA2-catalyzed oxidation of nicotine in the first step of this pathway are donated to the cytochrome *c* CycN^9^. Chromosomal deletion of the *cycN* gene arrests the natural pathway for nicotine degradation, severely impairing the strain’s ability to grow on nicotine (Fig. 1a). This is likely a consequence of the poor activity of NicA2 when using O_2_ as an electron acceptor, as the limited supply of carbon freed from nicotine breakdown in the Δ*cycN* strain is insufficient to sustain rapid growth^8,9^. *P*. *putida* S16 containing a Δ*cycN* mutation thus provides a genetic background that links bacterial growth directly to the ability of NicA2 to be oxidized by O_2_. We transformed a *P*. *putida* S16 Δ*cycN* strain with mutant libraries of *nicA2* and isolated variants that demonstrate substantially better growth on nicotine than wild-type *nicA2* (Fig. 1b). Essentially all variants of NicA2 that exhibited faster growth contained missense mutations that improved their O_2_-dependent catalytic rates (Supplementary Table 1). Multiple rounds of enrichment, random mutagenesis and reselection yielded variants with increasingly higher apparent *k*_cat_ values (Fig. 1c). The *k*_cat_ values of the encoded variants were positively correlated with growth in liquid culture, supporting our hypothesis that growth of the deletion strain is limited by the activity of NicA2 (Fig. 1d). We isolated variants with *k*_cat_ values as high as 1.25 s^−1^ for v417, a 189-fold improvement over the *k*_cat_ of 0.0066 s^−1^ of wild-type NicA2.

**Extended Data Fig. 1:**
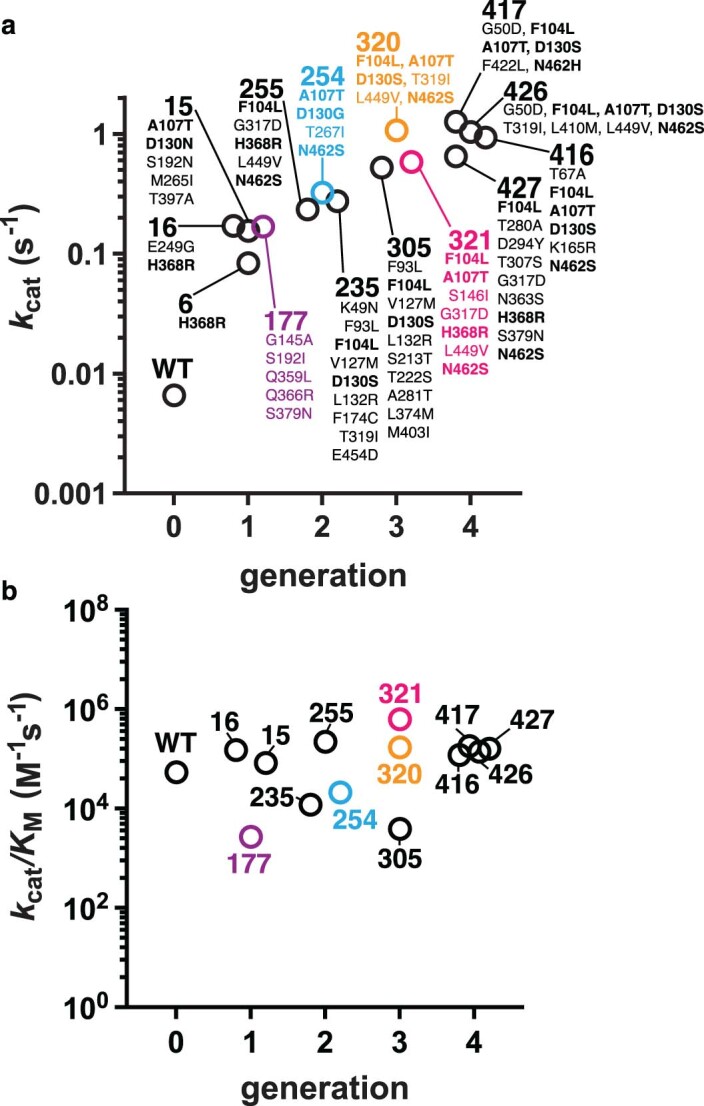
*k*_cat_/*K*_M_ is not reliably selected for under plate selection conditions. **a**, The top panel is a recreation of Fig. 1c with additional labels for comparison. Frequently mutated residues interrogated in Fig. 2 are bolded. **b**, *k*_cat_/*K*_M_ values for variants listed in Supplementary Table 1 plotted based on the generation of the selection they were isolated from. The color scheme holds between this figure and Fig. 1 of the main text. Source data

Improvements in *k*_cat_ of our variants often came at the cost of increased apparent *K*_m_ for nicotine (Supplementary Table 1). This is not surprising, as the 3 mM concentration of nicotine required for robust growth on the selective plates is well above the 114 nM *K*_m_ for nicotine that has been measured for the wild-type enzyme^12^. Thus, *K*_m_ is likely not under selective pressure. Despite this, the relatively larger increases in *k*_cat_ resulting from our selection led to NicA2 variants with up to a tenfold increase in *k*_cat_/*K*_m_ (Extended Data Fig. 1b).

**Fig. 2:**
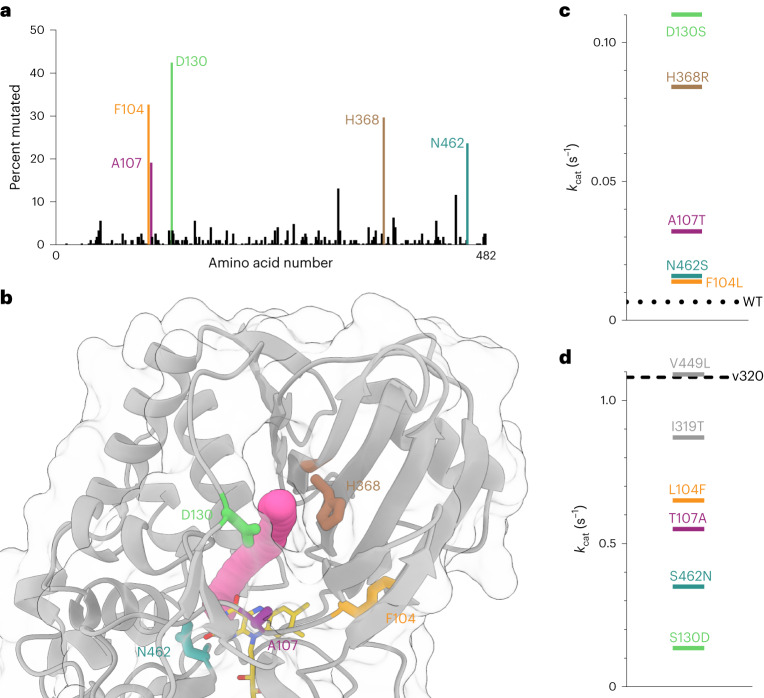
Mutations near FAD are critical for a gain in oxygen reactivity. **a**, One hundred and thirty-three variant sequences were isolated from our selection. The observed percentage of missense mutations at each amino acid location along the protein’s sequence is plotted. Note that these variant sequences are not independent because the iterative mutagenesis used to create new mutant libraries was done using a pool of higher-activity variants as a template and is therefore subject to a founder effect between different generations of the selection. The color scheme in **a** applies to the rest of the figure. **b**, The crystal structure of wild-type NicA2 (Protein Data Bank (PDB) ID 5TTJ) is displayed with a tunnel diameter of ~1.4 Å identified by CAVER simulation rendered in magenta^43^. **c**, *k*_cat_ values determined for single mutations in the background of wild-type NicA2. The dotted line indicates the *k*_cat_ value of wild-type NicA2. **d**, *k*_cat_ values determined for variants where mutations were removed from the background of NicA2 v320; each line represents the *k*_cat_ value corresponding to single mutations back toward the wild-type NicA2. Note the tenfold difference in scale from the plot shown in **c**. The dashed line indicates the *k*_cat_ value of NicA2 v320. The location of the amino acid positions L449 and T319 in the crystal structure can be seen in Extended Data Fig. 4. Source data

**Extended Data Fig. 2:**
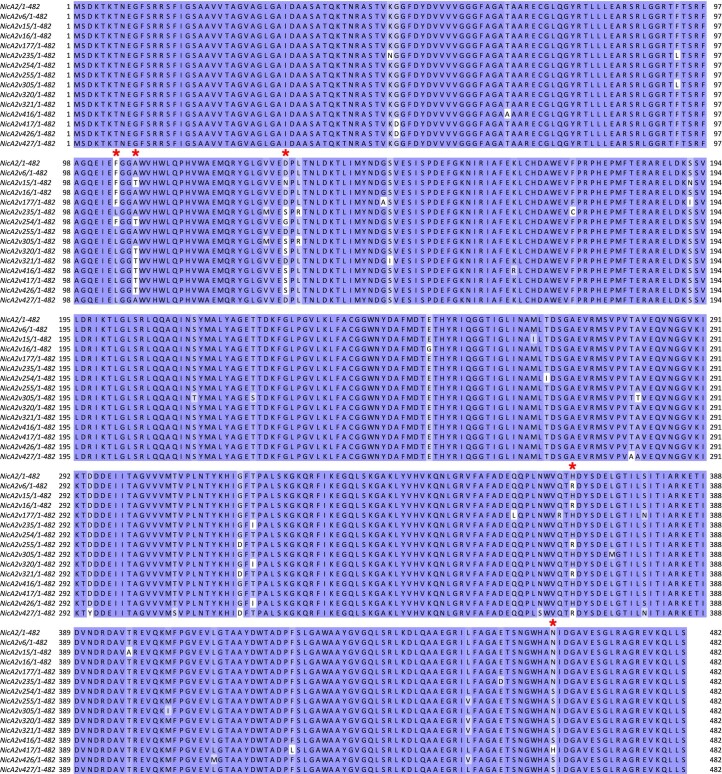
Alignment of NicA2 variants tested for steady-state kinetic activity. Red asterisks denote amino acid locations found to be mutated in greater than 15% of all sequences isolated from the selection. Sequences are colored for their degree of conservation using the BLOSUM62 score. Figure was prepared using Jalview^57^.

The majority of sequenced variants contained a mutation at one or more of the residues F104, A107, D130, H368 and N462 (Fig. 2a). These frequently mutated residues cluster near the *si*-flavin region (Fig. 2b). To determine if each of these mutations individually increase the catalytic activity of NicA2, we introduced the consensus substitution for each position into the background of wild-type NicA2 (Fig. 2c and Extended Data Fig. 2). The two single substitutions of greatest benefit to enzyme activity were D130S and H368R. These substitutions appear too distant to directly influence the chemical environment for the activation of O_2_ near flavin and may instead impact oxygen access to the protein interior.

**Extended Data Fig. 3:**
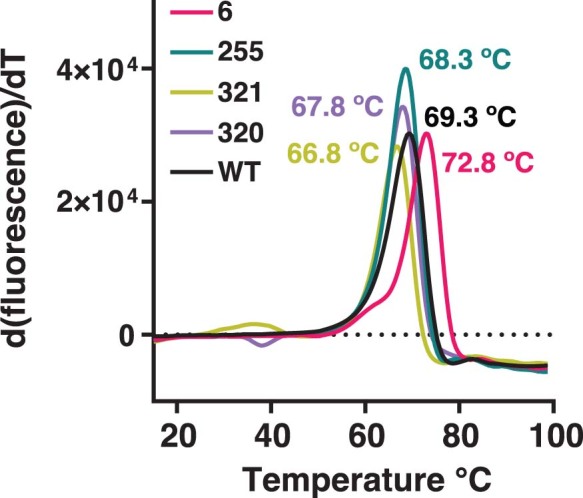
NicA2 wildtype and variants maintain a similar melting temperature. NicA2 proteins as listed in the figure were subject to the ThermoFAD melting assay to determine the temperature at which they unfolded, releasing their bound FAD. The melting temperature of each variant is displayed in its corresponding color. Displayed curves are the average of three replicates. Source data

The most active variants isolated from the selection require several of these common substitutions for robust activity (Extended Data Fig. 2). For example, NicA2 v320 contains the four mutations F104L, A107T, D130S and N462S and the less common T319I and L449V. To establish which of these substitutions were the most critical for the increase in turnover rate in this variant, we individually restored the wild-type amino acids at these positions and assessed the activity of the resulting variants. Of the six mutations in NicA2 v320, we found that the four highly enriched mutations proximal to the flavin (F104L, A107T, D130S and N462S) are most important for activity. The two more distant mutations (T319I and L449V) had less of an effect on enzyme activity (Fig. 2d). We next screened the thermostability of several of our variants and found that they had melting temperatures similar to that of wild-type NicA2 (Extended Data Fig. 3)^20^. Mutations are often destabilizing, so this result may seem surprising, but because our selection demanded increased activity destabilizing mutations were likely selected against^21^.

### NicA2 variants have markedly higher oxidase activity

**Fig. 3:**
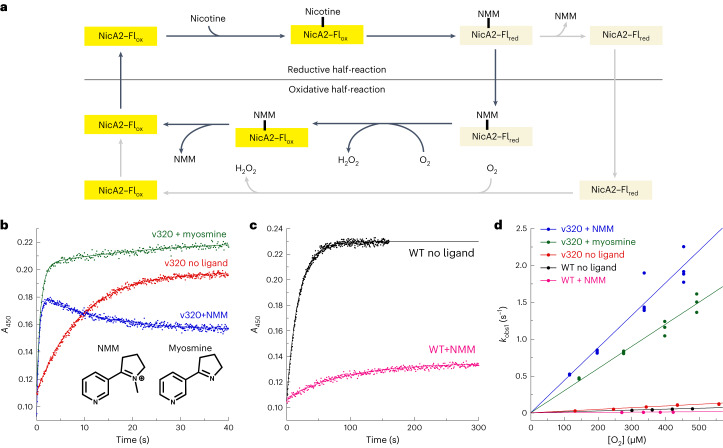
NicA2 variants are rapidly oxidized by O_2_. **a**, Schematic of the reaction of NicA2 with nicotine; Fl_ox_, oxidized flavin; Fl_red_, reduced flavin. **b**, Example traces for the stopped-flow reaction of reduced NicA2 v320 with O_2_ at a concentration of 450 μM. Raw traces for reactions at all concentrations of O_2_ can be seen in Supplementary Fig. 1a–h. The second phase of the reaction of NicA2 v320 in the presence of myosmine likely represents a subpopulation of the enzyme that is reacting with O_2_ in the ligand-free state (Supplementary Fig. 1i). Also shown are the chemical structures for NMM and myosmine. **c**, Example traces for the reaction of reduced wild-type NicA2 with O_2_ at a concentration of 450 μM. **d**, *k*_obs_ values for the first phase of oxidation are plotted against O_2_ concentration ([O_2_]) and fit to a line. The slopes of these lines define the bimolecular rate constants for oxidation by O_2_, which are presented in numeric form in Table 1. Source data

**Extended Data Fig. 5:**
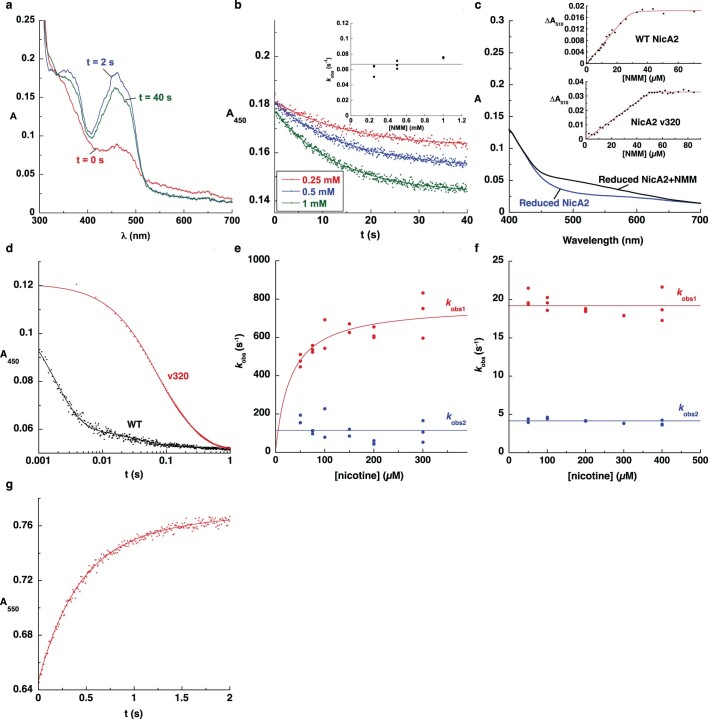
Binding and stopped flow reactions of NicA2 wildtype and v320. **a**, Visible absorbance spectra of select time points for the reaction of reduced NicA2 v320 in the presence of 1 mM NMM with O_2_ (see Fig. 3b of main text). **b**, Stopped-flow absorbance traces for the interaction between oxidized NicA2 v320 and NMM at several NMM concentrations. The inset shows the *k*_obs_ values of the reaction traces plotted against NMM concentration. Note that the *k*_obs_ value was 0.08 s^−1^ for the secondary decrease in absorbance observed for the reaction of reduced NicA2 v320 in the presence of 1 mM NMM with O_2_ (Fig. 3b of main text), which matches the *k*_obs_ value when oxidized v320 interacts with 1 mM NMM. **c**, Reduced NicA2 wildtype and v320 were titrated with NMM, monitoring absorbance at 510 nm to determine the binding coefficient. The inset shows the data points from the titration fit with the tight binding equation. **d**, Raw traces for the reactions of NicA2 wildtype and v320 with nicotine, both demonstrating biphasic traces. Note the logarithmic x-axis. Raw traces for reactions at all concentrations of nicotine can be seen in Supplementary Fig. 1j–l. **e**, *k*_obs_ values for the reaction of wildtype NicA2 are plotted against the concentration of nicotine. *k*_obs_ values from first phase was fit to a hyperbola, *k*_obs_ from the second phase was invariant to nicotine concentration. **f**, *k*_obs_ values for the reaction of NicA2 v320 are plotted against the concentration of nicotine, both were invariant to the nicotine concentration. **g**, The reaction of reduced NicA2 v320 with oxidized CycN was rapid, finishing in roughly 2 seconds similar to the reaction of wildtype NicA2^9^. Source data

NicA2 v320 had one of the highest *k*_cat_ values for O_2_-dependent nicotine degradation, so we selected it for detailed analysis. NicA2 v320 contains a spectrum of activating mutations in common with many of the other highest *k*_cat_ variants (Extended Data Fig. 2), so we reasoned that the results obtained for it should be representative. Stopped-flow experiments that monitored the reaction of reduced NicA2 v320 with O_2_ revealed that FADH_2_ oxidation is accelerated 157-fold in this variant relative to wild-type NicA2, but only when the reaction product NMM is bound (Fig. 3a and Table 1). The bimolecular rate constant for flavin oxidation by O_2_ (${k}_{{{\mathrm{ox}}}}^{{{\mathrm{O}}}_{2}}$) was 4,400 M^−1^ s^−1^ for NicA2 v320 in the presence of NMM, falling within the 10^3^–10^6^ M^−1^ s^−1^ range typically seen for bona fide flavin-containing amine oxidases^14^. However, the ${k}_{{{\mathrm{ox}}}}^{{{\mathrm{O}}}_{2}}$ was only 230 M^−1^ s^−1^ in the absence of NMM (Fig. 3b). This indicates that NMM binding to NicA2 v320 is necessary for the substantial enhancement we observe in the rate at which its flavin can be oxidized by O_2_. The opposite behavior occurs in wild-type NicA2. Here, the addition of NMM decreases ${k}_{{{\mathrm{ox}}}}^{{{\mathrm{O}}}_{2}}$ to 28 M^−1^ s^−1^ relative to the already low value of 120 M^−1^ s^−1^ that is observed in the absence of NMM (Fig. 3c). Reaction traces for oxidation of NMM-bound reduced NicA2 v320 by O_2_ display a second decrease in absorbance that occurs following flavin oxidation (Fig. 3b and Extended Data Fig. 5a). This decrease could be reproduced by simply mixing oxidized NicA2 v320 with NMM (Extended Data Fig. 5b), indicating that the decrease in absorbance in Fig. 3b is due to an interaction between oxidized NicA2 v320 and NMM after the flavin has been oxidized by O_2_. We previously observed a similar decrease in flavin absorbance after mixing oxidized wild-type NicA2 with NMM^9^. This change persisted under aerobic conditions, indicating that it is not due to NMM-triggered reduction of NicA2’s flavin. The decrease is not observed in the equivalent reaction trace between nicotine and wild-type NicA2 because flavin oxidation by O_2_ is slow enough to be rate limiting, and the wild-type enzyme oxidizes directly into the NMM-bound state with reduced flavin absorbance as a single kinetic event (Fig. 3c). Spectrophotometric titrations indicate that reduced wild-type and variant NicA2 bind NMM with nanomolar affinities (Extended Data Fig. 5c). We previously determined that NMM formed due to nicotine oxidation remains bound to wild-type NicA2 when it is oxidized by its natural electron acceptor CycN^9^. This appears to be the case for NicA2 v320 oxidation by O_2_ as well because the apparent rate constant for oxidation of NMM-bound NicA2 v320 at 250 µM O_2_ (ambient concentration) is 1.1 s^−1^, which matches the measured *k*_cat_. This indicates that the ${k}_{{{\mathrm{ox}}}}^{{{\mathrm{O}}}_{2}}$ value for the NMM-bound enzyme is rate limiting during turnover, at least for this variant. It also implies that our mutations improve the catalytic rate of the enzyme by increasing the rate at which NMM-bound NicA2 is oxidized by O_2_. NicA2 v321, which contains a somewhat different spectrum of activating mutations, yielded similar results (Supplementary Fig. 2 and Table 1).

**Table 1:** Kinetic properties of NicA2 enzymes

Enzyme	*k*_cat_	Ligand-free ${k}_{{{\mathrm{ox}}}}^{{{\mathrm{O}}}_{2}}$	NMM-bound ${k}_{{{\mathrm{ox}}}}^{{{\mathrm{O}}}_{2}}$	*k*_red,1_	*k*_red,2_
Wild type	0.0066 s^−1^	120 M^−1^ s^−1^	28 M^−1^ s^−1^	800 s^−1^	120 s^−1^
v320	1.1 s^−1^	230 M^−1^ s^−1^	4,400 M^−1^ s^−1^	19 s^−1^	4.1 s^−1^
v321	0.58 s^−1^	230 M^−1^ s^−1^	2,000 M^−1^ s^−1^	130 s^−1^	10 s^−1^

Substrate or product binding has been shown to accelerate flavin oxidation for other amine oxidases^14,22^. This acceleration is thought to be due to a positively charged product acting to stabilize the transient superoxide intermediate that occurs during flavin oxidation by O_2_. To test if the positive charge on the pyrrolidine nitrogen of NMM is responsible for accelerating the reaction of NicA2 v320 with O_2_, we measured ${k}_{{{\mathrm{ox}}}}^{{{\mathrm{O}}}_{2}}$ for reduced NicA2 v320 in the presence of the uncharged product analog myosmine. (Fig. 3b). The ${k}_{{{\mathrm{ox}}}}^{{{\mathrm{O}}}_{2}}$ value for myosmine-bound NicA2 v320 was 2,900 M^−1^ s^−1^, which is comparable to that of NMM-bound NicA2 v320, suggesting that the positive charge of NMM is not important for the enhanced reactivity of NicA2 v320 (Fig. 3b,d).

### Oxidation of nicotine is impaired in NicA2 variants

To investigate the reductive half-reaction, we then measured kinetics for the reaction of oxidized NicA2 v320 with nicotine under anaerobic conditions. These measurements allowed us to determine the impact of the mutations in this variant on its reaction with nicotine. The reaction, as monitored by following absorbance changes that accompany flavin reduction, occurred in two kinetically observable phases (*k*_red,1_ and *k*_red,2_), both of which appeared to correspond to flavin reduction based on the observed changes in absorbance (Extended Data Fig. 5d). We previously observed a similar biphasic behavior with wild-type NicA2, which we attributed to the two subunits of the NicA2 homodimer reacting with different rate constants^9^. The observed rate constants for both kinetic events in NicA2 v320 were invariant with nicotine concentration, suggesting that the binding affinity for nicotine is substantially lower than the lowest nicotine concentration we could use (50 μM) while maintaining pseudo first-order conditions. The two rate constants for this reaction with NicA2 v320 were 42- and 29-fold lower, respectively, than the corresponding values with wild-type NicA2, indicating that the mutations in NicA2 v320 negatively impacted the enzyme’s ability to react rapidly with nicotine (Extended Data Fig. 5e,f). The rate constant for the second phase of flavin reduction (4.1 s^−1^) of NicA2 v320 was only fourfold greater than the apparent rate constant for the reaction with O_2_ at ambient O_2_ concentrations. If any additional mutations similarly compromise the flavin reduction rate, then the reaction of NicA2 variants with nicotine will become rate limiting rather than the reaction with O_2_. This would explain why we did not see major improvements in the catalytic activity of variants from the fourth generation of selection; additional mutations that may have improved oxidation by O_2_ decreased reactivity with nicotine to the point where they did not have a selectable growth advantage. Mutations in NicA2 v320 did not have a major impact on its ability to transfer electrons to CycN, and reduced NicA2 v320 reacted as rapidly with CycN as wild-type NicA2 (Extended Data Fig. 5g).

### A change in tunnel accessibility enhances catalysis

**Fig. 4:**
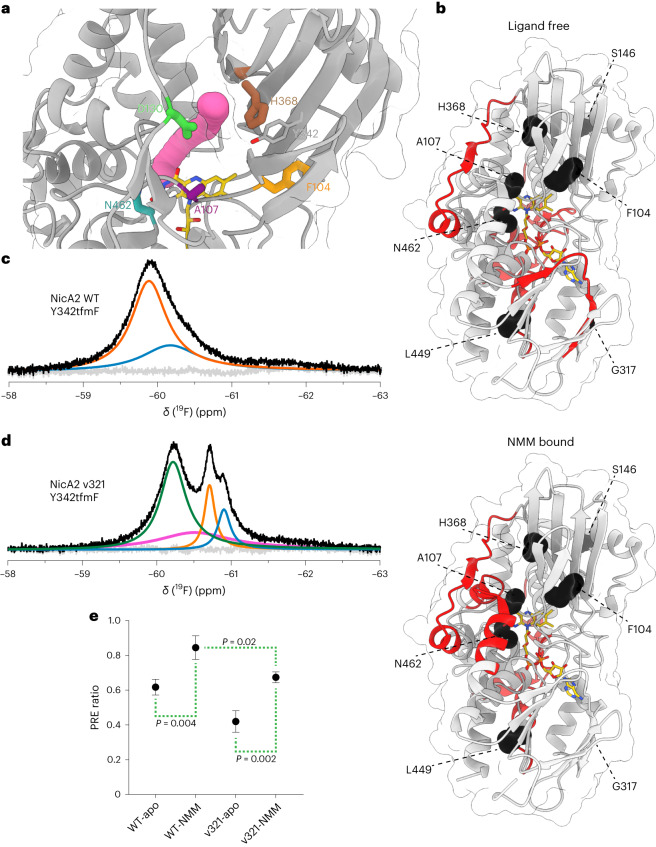
Wild-type NicA2 and mutant v321 populate distinct conformational landscapes. **a**, The crystal structure of wild-type NicA2 (PDB ID 5TTJ) is displayed with a tunnel ~1.4 Å in diameter identified by CAVER simulation rendered in magenta. FAD is rendered in yellow, Y342 is rendered in gray, and labeled frequently mutated residues are rendered in orange (F104), purple (A107), green (D130), brown (H368) and turquoise (N462). **b**, Differential HDX-MS data are shown on the structure of wild-type NicA2 under either a ligand-free or NMM-bound condition. Sections of the protein backbone that are highlighted in red demonstrate greater solvent exchange in v321 than in the wild-type protein. Interestingly, we also observed other areas of deprotection in NicA2 away from this tunnel region, suggesting that some additional structural rearrangements are also occurring. Residues that are mutated in NicA2 v321 are rendered as black spheres. **c**, ^19^F NMR spectrum of wild-type NicA2 with the Y342tfmF substitution. The black trace represents the raw data, the colored curves represent fits deconvoluted using decon1d^44^, and the gray trace represents residuals from the fit. **d**, ^19^F NMR spectrum of NicA2 v321 with the Y342tfmF substitution. **e**, PRE ratios for wild-type NicA2 and v321 in both apo- and NMM-bound states. A lower PRE ratio indicates increased accessibility to solvent. The mean is plotted with error bars that represent the s.d. of three replicates. Data were analyzed by one-way analysis of variance with a Tukey multiple comparisons post hoc test. Source data

**Extended Data Fig. 6:**
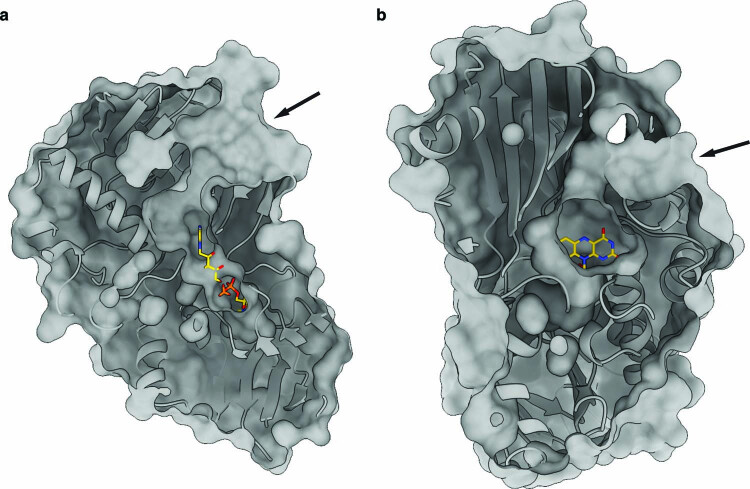
NicA2 wildtype has solvent exposed channels. **a**, The crystal structure of wildtype NicA2 (PDB 5TTJ) is rendered with the solvent accessible surface area. The arrow marks a solvent exposed channel into the active site of NicA2. **b**, A different view of wildtype NicA2. The arrow marks a cavity on the surface of the protein that approaches the active site.

We next wanted to understand the structural basis for the increased oxidation rate of NicA2 variants. Genuine flavoenzyme oxidases often contain tunnels within the protein structure for the guided diffusion of O_2_ into the protein core that houses the flavin cofactor. These tunnels can be a permanent feature evident from crystal structures^23^ or they can form transiently in the catalytic cycle, making them difficult to characterize experimentally^24^. Inspection of the solvent-accessible surface area in the crystal structure of wild-type NicA2 revealed several interconnected cavities that extend from the *si*-side of the FAD isoalloxazine moiety to the surface (Extended Data Fig. 6). One such tunnel is in a region that is rich with the mutations that we discovered that improve the oxidase activity of NicA2 (Fig. 4a). This led us to wonder if the observed gain in activity for NicA2 variants is at least partially due to structural perturbations that make this tunnel more accessible to O_2_.

**Extended Data Fig. 7:**
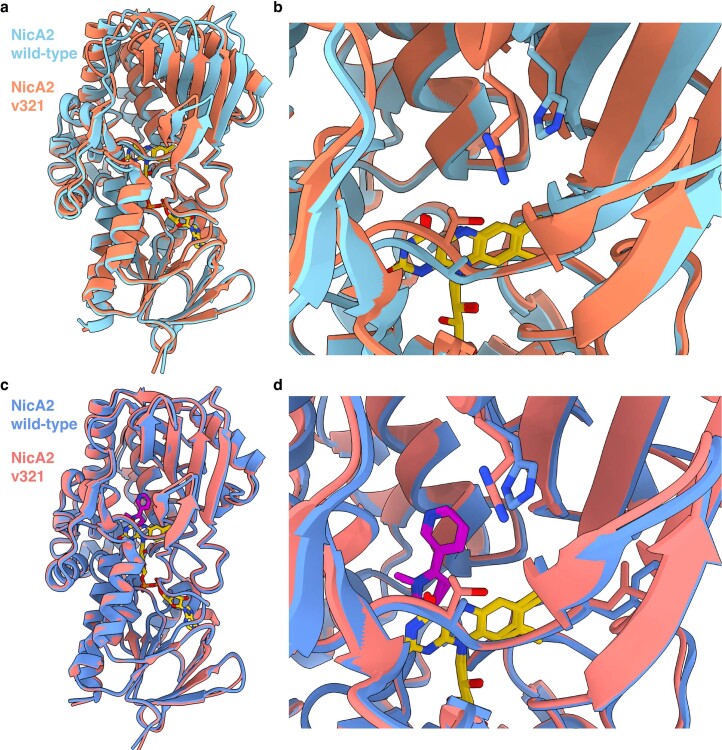
NicA2 wildtype and v321 have similar structures when bound by N-methylmyosmine. We first attempted to crystallize NicA2 v320, but none of our crystals diffracted to adequate resolution for structure determination. We were fortunate to obtain well diffracting crystals of NicA2 v321 in both the ligand-free and NMM-bound forms. The mutational changes and kinetic parameters of v320 and v321 are similar, so the structural perturbations seen for v321 are likely informative for those in v320 and other variants that contain similar changes. **a**, Unbound NicA2 wildtype (PDB: 5TTJ) and v321 enzyme structures were overlaid, aligned at their flavin binding domains. **b**, Overlay of the active sites of the enzymes with A107T and H368R mutations rendered. **c**, NicA2 wildtype and v321 enzyme structures bound to N-methylmyosmine were overlaid, aligned at their flavin binding domains. **d**, Overlay of the active sites with A107T and H368R mutations rendered. Wildtype enzyme is rendered in cyan/blue, v321 in orange/salmon, Flavin adenine dinucleotide in yellow, and N-methylmyosmine in magenta.

We were unable to solve structures of NicA2 v320 but were fortunate to obtain well-diffracting crystals of NicA2 v321 in both the ligand-free and NMM-bound forms. The ligand-free structures of the wild-type and v321 enzymes are somewhat different, with the crystallized conformation of ligand-free v321 closely mimicking the NMM-bound state of both wild-type and variant enzymes (Extended Data Fig. 7a,b). By contrast, the NMM-bound structures of v321 and wild-type NicA2 are very similar with an overall root mean squared deviation of <0.5 Å and no obvious difference that would explain our observed change in activity with O_2_ (Extended Data Fig. 7c,d).

**Extended Data Fig. 8:**
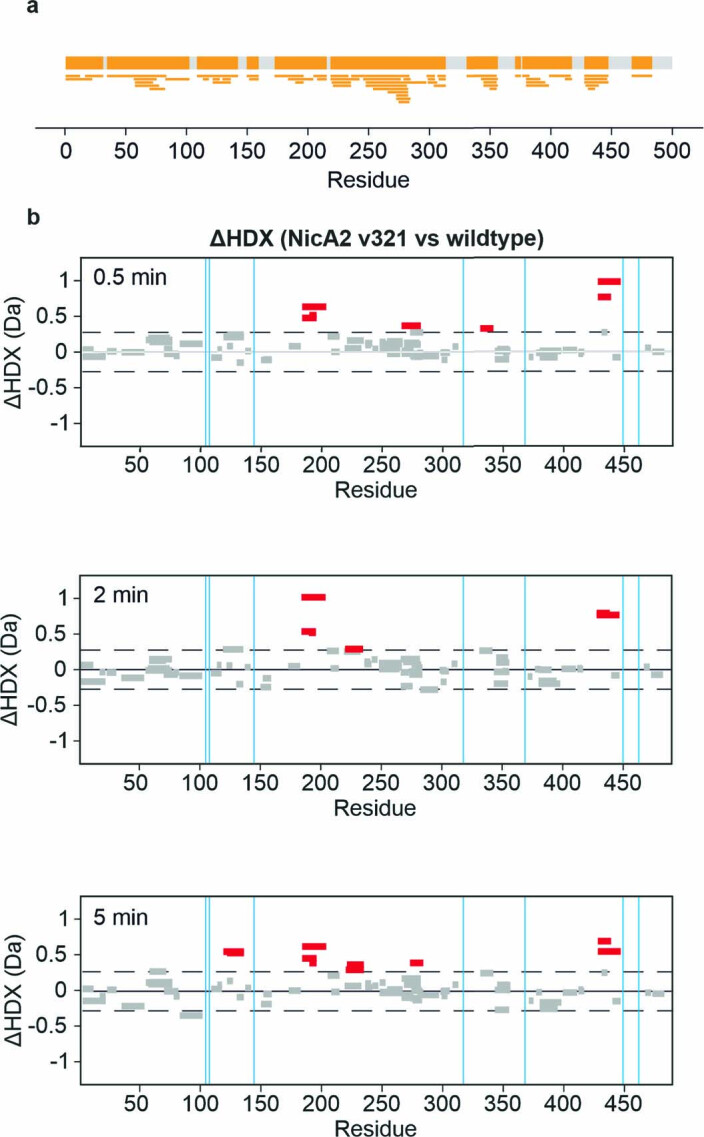
Differential HDX-MS analysis of NicA2 v321 vs wildtype. **a**, Peptide coverage map, showing the common peptides detected for wild-type and NicA2 v321. Peptides where the mutations are present are not included in these analyses. **b**, Woods’ plots showing the difference in deuterium uptake between peptides derived from wild-type or NicA2 v321 for the 0.5, 2, 5 min timepoints. Peptides that showed statistically significant deprotection in v321 are colored in red. No regions of protection from exchange were detected. The vertical thin blue lines represent the location of mutations between NicA2 wildtype and v321.

**Extended Data Fig. 9:**
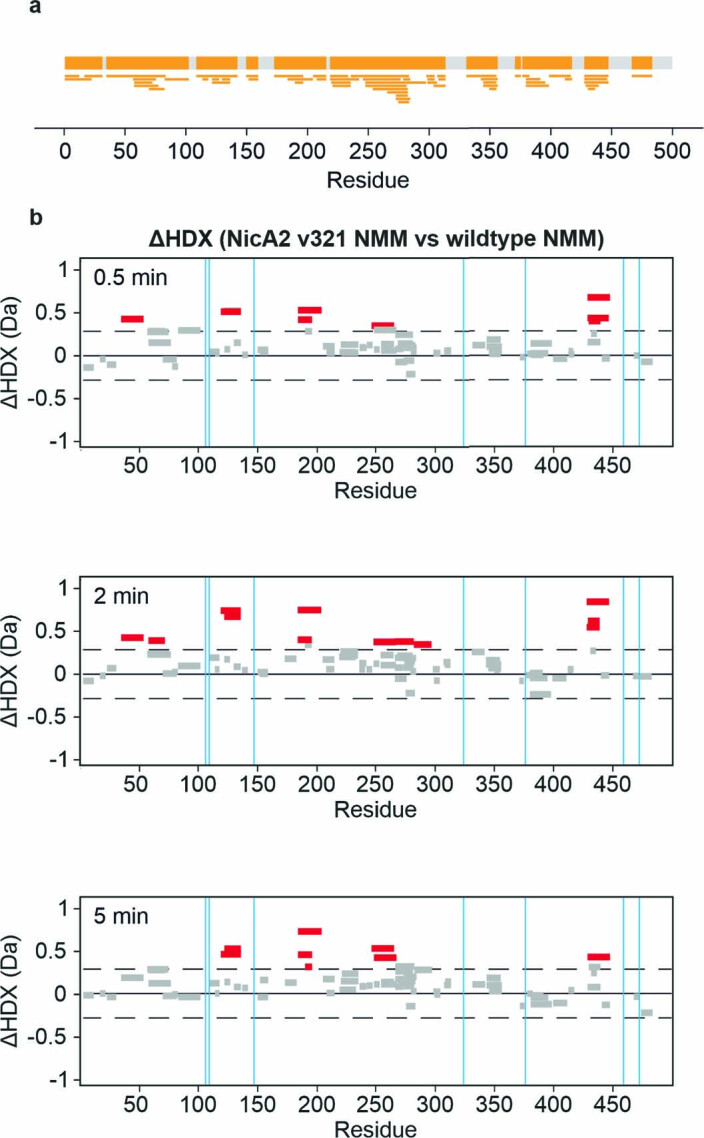
Differential HDX-MS analysis of NMM-bound NicA2 v321 vs wildtype. **a**, Peptide coverage map, showing the common peptides detected for wild-type and NicA2 v321. Peptides where the mutations are present are not included in these analyses. **b**, Woods’ plots showing the difference in deuterium uptake between peptides derived from wild-type or NicA2 v321 when bound to NMM for the 0.5, 2, 5 min timepoints. Peptides that showed statistically significant deprotection in v321 are colored in red. No regions of protection from exchange were detected. The vertical thin blue lines represent the location of mutations between NicA2 wildtype and v321.

Enzymes that have a buried active site need to facilitate substrate migration for catalysis to occur efficiently. The observed increase in the oxidation rate of NMM-bound NicA2 v321 raises an intriguing possibility that the mutations in this variant alter the transport pathway of O_2_. To assess the presence and dynamicity of oxygen tunnels in NicA2, we used the label-free method differential hydrogen–deuterium exchange mass spectrometry (HDX-MS). Differential HDX-MS provides information on the solvent accessibility and dynamics of backbone amide protons in proteins. Analyzing differences in deuterium uptake between wild-type NicA2 and v321, we observed an increase in deuterium incorporation in NicA2 v321 compared to wild-type (Extended Data Figs. 8 and 9) in both the ligand-free and NMM-bound states. Increased uptake is observed in the area surrounding the putative tunnel in v321, suggesting that it is more accessible to solvent (Fig. 4b). This is consistent with our hypothesis that mutations in this region facilitate O_2_ transport to the active site.

### Mutations in NicA2 shift conformation of a putative O_2_ tunnel

To further elucidate the chemical environment of this tunnel, we used an NMR site-specific probe, as this approach can provide structural and kinetic information on transient events in enzyme action^25^. Given the large size of the NicA2 homodimer (110 kDa) and the subtle conformational changes we wish to probe, we decided to use one-dimensional ^19^F NMR. Fluorine serves as an exquisitely sensitive structural probe for side chain dynamics. Observation of anything other than a single sharp peak in the ^19^F NMR spectrum indicates conformational heterogeneity in the local environment of the fluorine nucleus. We used amber codon suppression to site-specifically incorporate the unnatural amino acid 4-trifluoromethyl-l-phenylalanine (tfmF)^26^ in place of Y342, a residue that is located adjacent to the tunnel in the wild-type protein (Fig. 4a). We judged that this substitution is unlikely to block the opening of the tunnel and found that it does not impact enzyme function or stability (Supplementary Fig. 3).

We observed a single broad signal in the ^19^F spectrum of wild-type NicA2 at −59.8 ppm. This could be deconvoluted into two highly overlapping peaks indicative of conformational motion in the intermediate-to-slow exchange regime in the interior of the tunnel (Fig. 4c). The ^19^F spectrum of NicA2 v321 was strikingly different and was composed of a broad upfield-shifted major peak at −60.2 ppm and three highly overlapping downfield minor peaks (Fig. 4d). By contrast, at a control substitution in a position that lies far from the tunnel region, we observed single narrow peaks in both wild-type NicA2 and v321 (Supplementary Fig. 4a). Wild-type NicA2 and v321 also demonstrated a nearly identical elution profile in size-exclusion chromatography (Supplementary Fig. 5). These data suggest that the multiple peaks we detect with a single tfmF incorporated at Y342 cannot be explained by global structural perturbation. In summary, the ^19^F NMR spectra of the two ligand-free NicA2 molecules demonstrate a higher degree of conformational heterogeneity in the proposed oxygen tunnel in NicA3 v321 than in the wild-type protein.

**Extended Data Fig. 10:**
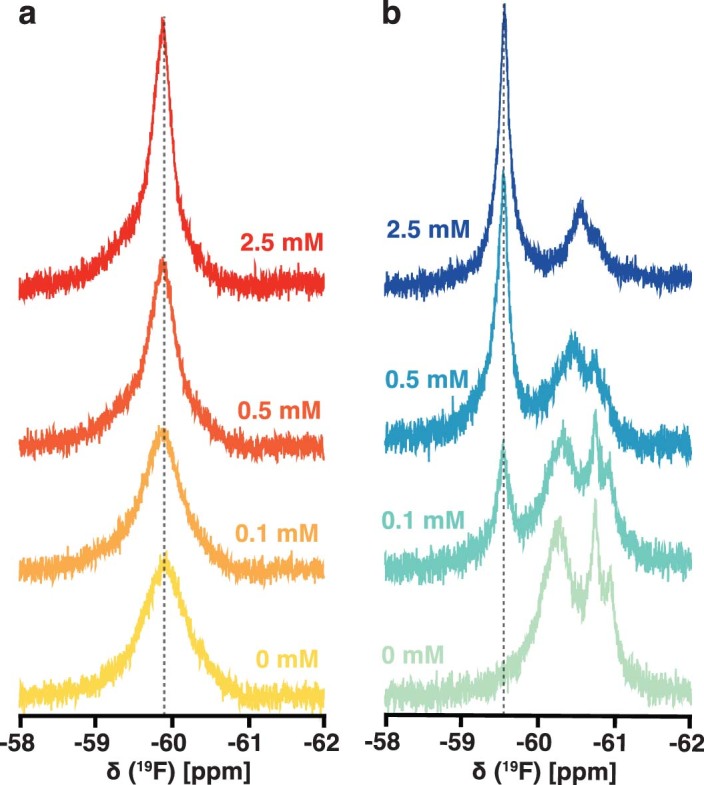
NicA2 wildtype and v321 titrations with NMM. **a**, The signal for wildtype NicA2 Y342tmfF titrated with 0, 0.1, 0.5, and 2.5 mM NMM narrows with increasing NMM, indicating a more restricted sampling of local conformations. **b**, NicA2 v321 Y342tmfF titrated with NMM of the same concentrations as labeled, showing collapse into a single population. At increasing molar ratios, NMM yields a narrow major peak whose intensity increases with a concomitant decrease in the intensity of the apo- state peaks. Thus, NMM binding ‘pulls’ the distinct apo- state sub-ensembles that are in slow exchange into a single major conformation, one that presumably resembles the crystallized conformation of the NMM-bound NicA2 v321 complex. NMM, N-methylmyosmine. Source data

Our stopped-flow experiments demonstrated that NicA2 v321 has an increased rate of oxidation compared to the wild-type enzyme, but only when it is bound by the reaction product NMM. To explore the structural basis for this finding, we compared the NMM-bound state of wild-type NicA2 and that of v321. The addition of NMM did not cause any notable chemical shift perturbation in wild-type NicA2, although the peak narrowed, suggesting that ligand binding may restrict conformational fluctuations in the tunnel (Extended Data Fig. 10a). After addition of NMM to NicA2 v321, we observed a substantial downfield chemical shift in the major peak of the enzyme, possibly due to conformational rearrangement in the tunnel region (Extended Data Fig. 10b). We confirmed that these conformational changes are relevant to catalysis by observing a similar spectrum after the addition of nicotine to the enzyme to achieve the NMM-bound, reduced enzyme state that was rapidly oxidized by O_2_ in our kinetic experiments (Supplementary Fig. 4b). Therefore, observations made using the NMM-bound oxidized enzyme report on the conformation of the enzyme relevant for the oxidation step.

Paramagnetic relaxation enhancement (PRE) is widely used to obtain long-range distance restraints in NMR spectroscopy^27^. When a soluble paramagnetic reagent, such as 4-hydroxy-2,2,6,6-tetramethyl-piperidine-1-oxyl (TEMPOL), is added to the solvent, the extent of paramagnetic broadening depends on solvent accessibility of fluorine. We decided to use solvent accessibility as a proxy to ascertain access of O_2_ to the proposed tunnel. In the presence of TEMPOL, we observed line broadening in the major peaks in both the unbound wild-type and mutant NicA2 forms (59.8 and 60.2 ppm, respectively), suggesting that these peaks represent accessible conformations (Supplementary Fig. 6). The major populations of both wild-type NicA2 and NicA2 v321 appear less accessible to solvent when bound by NMM than when ligand free (Fig. 4e). However, in both the ligand-free and NMM-bound states, the predominant conformations of NicA2 v321 have a more accessible tunnel than the respective states of the wild-type protein, in agreement with the HDX-MS data.

### NicA2 v321 effectively degrades plasma nicotine in vivo

**Fig. 5:**
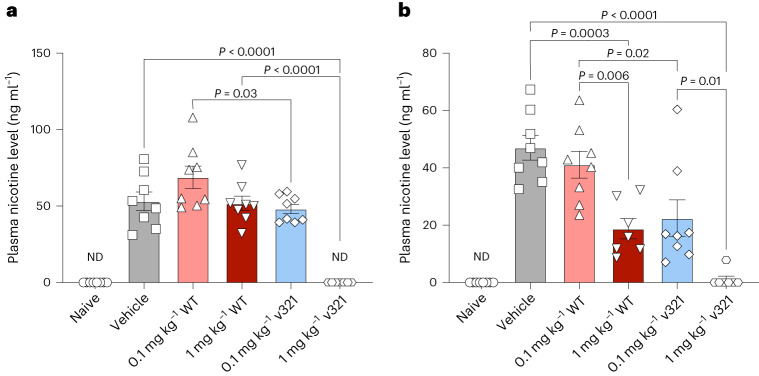
NicA2 v321 degrades plasma nicotine in rats. Adult Wistar rats (*N* = 48, equal numbers of males and females) were exposed to nicotine (free base) at a concentration of 3.15 mg per kg (body weight) per d to achieve a chronic blood nicotine level of 50 ng ml^−1^ or were administered 0.9% saline for 7 d via osmotic minipump. Rats were injected with 0.1 mg per kg (body weight) wild-type NicA2, 1 mg per kg (body weight) wild-type NicA2, 0.1 mg per kg (body weight) NicA2 v321 or 1 mg per kg (body weight) NicA2 v321 at the time of minipump implantation and again every 48 h. Blood was collected from animals at 1 and 5 d after minipump implantation, and plasma nicotine levels were analyzed by liquid chromatography–MS (LC–MS). Because we did not observe differences between sexes, the data reflect both sexes combined. **a**, Plasma nicotine levels after 1 d of nicotine exposure (24 h after injection of either vehicle or NicA2 treatments); additional statistical significance values not shown on the figure: for naive versus vehicle, 0.1 mg per kg (body weight) wild type, 1 mg per kg (body weight) wild type and 0.1 mg per kg (body weight) NicA2 v321, all *P* < 0.0001; for 1 mg per kg (body weight) NicA2 v321 versus vehicle, 0.1 mg per kg (body weight) wild type, 1 mg per kg (body weight) wild type and 0.1 mg per kg (body weight) NicA2 v321, *P* < 0.0001; *n* = 8 per treatment group and *n* = 7 for the 1 mg per kg (body weight) NicA2 v321 group; ND, s.e. not determined. **b**, Plasma nicotine levels after 5 d of nicotine exposure (24 h after injection of either vehicle or NicA2 treatments); additional statistical significance not shown on the figure: naive versus vehicle, 0.1 mg per kg (body weight) wild type, *P* < 0.0001; naive versus 1 mg per kg (body weight) wild type, *P* = 0.03; naive versus 0.1 mg per kg (body weight) NicA2 v321, *P* = 0.004; 1 mg per kg (body weight) NicA2 v321 versus 0.1 mg per kg (body weight) wild type, *P* < 0.0001; *n* = 8 per treatment group and *n* = 7 for the 1 mg per kg (body weight) NicA2 v321 and 1 mg per kg (body weight) wild type groups. Data were analyzed by one-way analysis of variance with Tukey’s multiple comparisons post hoc tests. The mean is plotted with error bars that represent the s.e. Source data

To assist in the cessation of smoking, a NicA2 variant must degrade nicotine effectively in vivo. To compare the efficacy of NicA2 v321 to that of wild-type under clinically relevant conditions, we exposed rats to chronic nicotine levels similar to those found in smokers and administered NicA2 enzymes to assess their nicotine degradation capabilities. We conjugated NicA2 v321 and wild-type NicA2 to an albumin binding domain (ABD) for enhanced stability in the blood before injection^4^. NicA2 v321 and wild-type NicA2 decreased blood nicotine levels in a dose-dependent manner (Fig. 5). NicA2 v321 was tenfold more efficacious at degrading nicotine than the wild-type protein, as demonstrated by the near identical performance of 1 mg per kg (body weight) wild-type enzyme and 0.1 mg per kg (body weight) NicA2 v321. This finding mirrors the approximately tenfold higher specificity constant (*k*_cat_/*K*_m_) of NicA2 v321 than the wild-type protein (Supplementary Table 1). The highest dose of NicA2 v321 (1 mg per kg (body weight)) had undetectable plasma nicotine levels.

## Discussion

A unifying framework that defines how flavoproteins suppress or enable their reactivity with O_2_ has long been sought^17^. FADH_2_ free in solution can be oxidized by O_2_ with a bimolecular rate constant of 250 M^−1^ s^−1^ (ref. ^13^). When FADH_2_ is bound to an enzyme, the oxidation rate may be profoundly suppressed or accelerated to between 2 and 10^6^ M^−1^ s^−1^ (ref. ^15^). NicA2 is curiously discerning as to its preference for electron acceptors. Unlike the vast majority of flavin-dependent amine oxidase family members, NicA2 exhibits a very limited capacity to react with O_2_. The other notable exception to this paradigm is the recently characterized pseudooxynicotine amine dehydrogenase, which also uses CycN as its in vivo electron acceptor^28^. Donating electrons to CycN appears to be an evolutionary adaptation by which *P. putida* S16 can budget valuable reducing equivalents freed during the catabolism of nicotine instead of producing H_2_O_2_ by the reduction of O_2_^9,28^. It is not clear if this adaptation is widespread in nature. Other organisms such as *Arthrobacter nicotinovorans* use the alternative pyridine pathway for the catabolism of nicotine and contain homologous flavoenzymes that react readily with O_2_^29^. The structural similarity between NicA2 and these O_2_-utilizing flavin-dependent amine oxidases is undeniable^10^, which makes NicA2 an attractive model with which to explore the control of O_2_ reactivity in flavoenzymes.

Here, we developed a genetic selection strategy that links growth of *P. putida* S16 to the activity of NicA2 in the absence of its physiologic electron acceptor CycN. We used this selection to isolate variants of NicA2 with a substantial improvement in their rate constant for oxidation by O_2_ and a tenfold increase in *k*_cat_/*K*_m_. Multiple mutations are required for the largest increases in activity. A previous study demonstrated a threefold increase in *k*_cat_/*K*_m_ from a single substitution in NicA2-A107R^7^. This position was also heavily substituted in our most active variants, although we found A107T to be the predominant change. The relative bulk of the A107R mutation may impact solvent accessibility and tunnel dynamics, like we have observed in our active variants, explaining its effect on activity.

NicA2 variants only exhibit increased rate constants for oxidation by O_2_ when bound by the reaction product NMM or its uncharged analog myosmine. That the positive charge on NMM is not required for this effect is surprising, as the presence of charge itself has previously been observed to stabilize the transition state for oxidation by O_2_, facilitating catalysis^14,22^. NMM binding may instead shift NicA2 variants toward a catalytically competent conformation. Alternatively, NMM could create a suitable hydrophobic interface near the flavin for the reaction of O_2_, as has been demonstrated to be important for some other flavoenzymes^30–32^. Because NMM remains bound during CycN-dependent catalysis^9^ and CycN appears to inhabit a binding site distinct from the location of our mutations^28,33^, it is not surprising that the oxidation rate with CycN is unimpaired in our variants.

One way by which flavoenzymes may modulate oxidation of FADH_2_ is by changing the accessibility of the protein interior to O_2_. Destabilizing a protein could improve oxygen permeability due to increased molecular breathing. However, our most effective variants exhibit remarkably unaltered thermodynamic stability. Dissolved oxygen may also penetrate enzymes using predefined oxygen channels^24,34–38^. Obstruction or modification of these channels can lead to a decrease in oxidase activity, which supports a direct role of channels in bringing O_2_ to the active site^34,35,38^. We observe that compared to the wild type, NicA2 v321 appears to have greater exposure to solvent in a mutation-rich region encompassing a putative O_2_ tunnel. This tunnel is also more conformationally heterogenous in the ligand-free condition of v321 than it is in the wild type. After NMM binding, NicA2 v321 undergoes major rearrangements in the tunnel region, as indicated by the collapse and substantial downfield shift of fluorine resonances in our ^19^F experiments. We further demonstrate that this new conformational state is overall more solvent accessible in NicA2 v321 than in wild-type NicA2. This may indicate that the mutations encoded in NicA2 v321 allow greater diffusion of O_2_ to flavin in this catalytically competent state. This could, at least partially, explain its increased oxidation rate.

Nicotine dependence is a major health problem, and smoking is responsible for one in ten deaths worldwide^39^. Nicotine is highly addictive, and greater than 95% of people who attempt to quit without specific medical intervention relapse within one year (refs. ^1,40^). Even with currently available therapies, the relapse rate remains stubbornly high at ~80% (ref. ^41^). Treatment with NicA2 provides an exciting direction to neutralize the psychoactive effect of nicotine. Ideally NicA2 would degrade most of the nicotine absorbed when smoking before it is able to reach the tissues upon which it acts. When inhaling nicotine, it takes only about 7 s for it to reach the brain^42^. Given the slow catalytic rate of wild-type NicA2, an extremely large dose of around 10–70 mg kg^−1^ is necessary to degrade nicotine in this short time frame. We have succeeded in isolating variants of NicA2 that are much more capable of reacting with O_2_ than the wild-type enzyme (${k}_{{{\mathrm{ox}}}}^{{{\mathrm{O}}}_{2}}$ of 28 M^−1^ s^−1^ when bound by NMM). Specifically, our variants, with ${k}_{{{\mathrm{ox}}}}^{{{\mathrm{O}}}_{2}}$ values of up to 4400 M^−1^ s^−1^, are much closer to the rate constants for oxidation exhibited by other flavin oxidases. We have thus come a considerable distance in restoring the oxidase activity of this enzyme implied by its designated enzyme family. NicA2 v321 is active in vivo at a tenfold higher efficacy than wild-type NicA2 and can maintain blood nicotine concentrations at undetectable levels. This increase in activity is a critical step in the development of an efficacious enzymatic therapy. Further investigation of these improved activity variants applied to addiction cessation models will be necessary to evaluate their clinical potential.

## Methods

### Strains and culture conditions

*P. putida* S16 was obtained from ATCC (BAA-2546). Generation of *P. putida* S16 Δ*cycN* was detailed in a previous study^9^. Bacterial culture was performed in lysogeny broth (LB) medium for general maintenance and growth of strains. Antibiotic selection was maintained using 100 μg ml^−1^ kanamycin or 25 μg ml^−1^ gentamicin for pET28 and pJN105 plasmids, respectively. Nicotine liquid medium was based on M9 minimal medium and included 6 g liter^−1^ Na_2_HPO_4_, 3 g liter^−1^ KH_2_PO_4_, 1 g liter^−1^ ammonium chloride, 1 mM MgSO_4_, 0.1 mM CaCl_2_, 1× trace metals (Teknova, T1001), 1 μg ml^−1^ thiamine and 0.5 g liter^−1^ nicotine. Nicotine selection plates were made with the same recipe as the liquid medium but instead used 1 g liter^−1^ nicotine, and 15 g liter^−1^ agarose was added (Thermo Fisher Scientific). Protein expression medium (PEM) consisted of 12 g liter^−1^ tryptone, 24 g liter^−1^ yeast extract, 50.4 g liter^−1^ glycerol, 2.13 g liter^−1^ K_2_HPO_4_ and 12.54 g liter^−1^ KH_2_PO_4_. *Escherichia coli* BL21 (DE3) cells were used for protein expression.

### *nicA2* variant library production

A high diversity library of *nicA2* variants containing ~10^8^ independent clones with an average mutation density of 8.9 nucleotide mutations per gene was obtained from Genewiz and cloned into the arabinose-inducible pJN105 vector^45^. This library was used for the first generation of selection. Later generations were obtained using serial error-prone PCR of pooled higher-activity variants from the previous rounds of selection. Briefly, error-prone PCR was performed using the Mutazyme II kit (Agilent) according to the manufacturer’s guidelines with primers P01/P02. We routinely used 100 ng of pooled variants as the template for the reaction. PCR products were run on an agarose gel, and the *nicA2* band was purified with a gel extraction kit (Macherey-Nagel). This mutagenized band was then used for a MEGAWHOP reaction onto pJN105-*nicA2* template as follows^46^. We prepared 400 μl of a Phusion PCR (Thermo Fisher) reaction with 400 ng of pJN105-*nicA2* wild type as the template and 1,500 ng of mutagenic PCR product as the megaprimer. PCR products were digested with DpnI for 3 h before ethanol precipitation with Pellet Paint (Sigma) additive, resuspension in 5 μl of double-distilled water and transformation into MegaX DH10b electrocompetent cells (Thermo Fisher). The 100-μl cell stocks were split into 25-μl volumes, and each was electroporated at 2,000 V in a 0.1-mm cuvette. Each electroporation reaction was resuspended in 1 ml of SOC medium and recovered with shaking at 37 °C for 1 h. Serial dilutions were plated onto LB–gentamicin plates to determine the size of the resulting libraries. Recovered cells were subcultured into LB with gentamicin and grown overnight, and their encoded plasmids were isolated (QIAprep Spin Miniprep kit, Qiagen) to generate the pJN105-*nicA2* libraries used in the selection. Homemade libraries ranged from 10^5^ to 10^6^ unique sequences in size.

### Selection of NicA2 variants with enhanced activity

The selection of NicA2 variants was performed using nicotine agar plates containing 1 g liter^−1^ nicotine. *P. putida* S16 Δ*cycN* was grown overnight at 30 °C. The next day, 0.25 ml of overnight culture was subcultured into 5 ml of fresh LB and grown for 3–4 h before being spun down and washed three times with 4 ml of ice-cold 300 mM sucrose. After these washes, cells were resuspended in 80 μl of 300 mM sucrose. Selection libraries in pJN105 were added to this concentrated cell stock and electroporated at 2,500 V (Electroporator 2510, Eppendorf). Next, 1 ml of SOC medium was added, and the cells were allowed to recover shaking at 30 °C for 1 h.

These strains that had been transformed with a mutagenized library were washed with M9 salts to remove any residual nutrient sources before being serially diluted and plated onto nicotine agar plates. Plates were supplemented with 25 μg ml^−1^ gentamicin as well as arabinose concentrations between 0.01 and 0.001% (wt/vol) as an inducer for NicA2 expression, with the lower concentrations inducing less NicA2 expression and thus providing a more stringent selection for nicotine degrading capability. Nicotine selection plates were incubated at 30 °C for 2–8 d until colonies appeared. The largest colonies were picked and restreaked onto nicotine agar plates for purification. Single colonies were picked from these streakouts and were then evaluated for activity in a plate reader-based secondary screen (see ‘Plate reader growth assay’) to determine which variants had the fastest rate of growth. The top performers from this secondary screen were sequenced and subcloned via a MEGAWHOP reaction^46^ into pET28a-His-SUMO for purification and analysis, as detailed below.

### Plate reader growth assay

Strains of *P. putida* S16 were streaked onto LB or nicotine agar plates to obtain single colonies. Strains containing NicA2 variants in the pJN105 plasmid background were maintained under 25 μg ml^−1^ gentamicin selection. Three distinct colonies were picked as biological replicates and grown in LB medium with shaking at 30 °C overnight. The next day, 2 μl of stationary-phase culture for each replicate was transferred to 200 μl of nicotine medium supplemented with 0.001% (wt/vol) arabinose in a 96-well plate. A Breathe-Easy sealing membrane plate cover (Sigma) was applied. The plate was set to shake with 2-mm amplitude at 30 °C, and absorbance was monitored at 600 nm in a Tecan M200 plate reader for 4 d. For each replicate, the growth rate for every 12-h window was estimated from changes in optical density at 600 nm (OD_600_) using linear regression, and mean growth rate was estimated from the replicates for each genotype.

### Purification of NicA2 variants for crystallography and stopped-flow assays

A pET28a-His-SUMO-*nicA2* plasmid was transformed into *E. coli* BL21 (DE3) cells for expression. Overnight cultures were diluted into 2–3 liter of PEM and grown to an OD_600_ of ~0.8 before being transferred to a 20 °C shaker and induced with 100 μM IPTG overnight. The following day, cultures were spun down, and cell pellets were stored at −20 °C before use. Cells were lysed by sonication at 4 °C in 50 mM Tris-HCl, 400 mM NaCl, 15 mM imidazole and 10% glycerol (pH 8.0; lysis buffer) with DNase I and cOmplete protease inhibitor cocktail. The lysate was cleared by spinning twice at 30,000*g* and 4 °C for 30 min. The clarified supernatant was then loaded onto three 5-ml HisTrap columns preequilibrated in lysis buffer. Columns were washed with 20 ml of lysis buffer and 20 ml of lysis buffer supplemented to 20 mM imidazole, and protein was eluted in lysis buffer supplemented to 0.5 M imidazole. NicA2 was dialyzed into 40 mM Tris-HCl (pH 8.0) and 0.2 M NaCl in the presence of the protease ULP-1 to cleave the His-SUMO tag. Dialyzed, cleaved protein was subsequently passed back over the HisTrap columns to remove the tag. Protein was then diluted into 25 mM Tris (pH 8.5) and loaded onto three HiTrap Q columns equilibrated in the same buffer. NicA2 was eluted using a linear salt gradient to 1 M NaCl. Fractions containing NicA2 were then concentrated before a final gel filtration over a HiLoad Superdex 200 column equilibrated in 40 mM HEPES and 100 mM NaCl (pH 7.4). Purified protein was supplemented with glycerol to 10% before being flash-frozen in liquid nitrogen and stored at −80 °C until use (Supplementary Fig. 7).

### Purification of NicA2 variants for steady-state reactions

NicA2 variants were PCR amplified from pJN105 vectors isolated from the selection using primers P01/P02. PCR products were gel extracted and used as megaprimers in a MEGAWHOP reaction onto template pET28a-His-SUMO-*nicA2* to create expression constructs. The MEGAWHOP reaction was digested with DpnI for 2–4 h before being transformed into *E. coli* 10b to isolate variants, which had their sequences confirmed by Sanger sequencing.

pET28a-His-SUMO-*nicA2* variant plasmids were transformed into *E. coli* BL21 (DE3) for expression. Overnight cultures were diluted into 1 liter of PEM and grown until an OD_600_ of ~0.8 before being transferred to a 20 °C shaker and induced with 200 μM IPTG overnight. The next day, samples were spun down, and the cell pellet was stored at −20 °C before purification. Cell pellets were resuspended in 50 mM sodium phosphate (pH 8.0), 100 mM NaCl and 10% (wt/vol) glycerol lysis buffer supplemented with phenylmethylsulfonyl fluoride to 1 mM, lysozyme to 1 mg ml^−1^ and benzonase nuclease to 0.5 U ml^−1^. These samples were sonicated on ice for 5 min and spun at 25,000*g* for 30 min. The supernatant was removed and added to 5 ml of Ni-NTA beads equilibrated in lysis buffer. The supernatant and bead mixture was set to rotate at 4 °C for 2 h before being washed with at least three column volumes of lysis buffer. Proteins were eluted with lysis buffer supplemented with imidazole to 500 mM. The eluate was set to dialyze overnight at 4 °C to dilute imidazole in the presence of ULP-1 protease. The next day, samples were reloaded onto Ni-NTA beads and passed through. Cleaved His-SUMO tag and ULP-1 protease remained on the column, while NicA2 variants were eluted in dialysis buffer. Proteins were concentrated and flash-frozen before being stored at −80 °C (Supplementary Fig. 7).

### Steady-state kinetic assays

All experiments were performed in 40 mM HEPES (pH 7.4), 100 mM NaCl and 10% (wt/vol) glycerol at 22 °C. The concentration of NicA2 was determined using the absorbance of bound FAD cofactor with an extinction coefficient at 450 nm of *ε* = 11,300 M^−1^ cm^−1^ (ref. ^9^). Purified NicA2 with a flavin concentration between 100 nM and 2 μM, depending on the variant, was mixed 1:1 with solutions of nicotine typically ranging from 5 to 1,000 μM and transferred to a 1-cm cuvette. The concentration of the substrate was always at least 50 times greater than that of the enzyme. Change in absorbance at 280 nm was monitored over time, and the linear portions of the resulting curves corresponding to no greater than 10% of the total reaction were fit to determine the initial velocity of the reaction. This value was divided by the difference between extinction coefficients of nicotine and NMM at 280 nm (*ε* = 2,914 M^−1^ cm^−1^, as determined by measuring pure samples of each substance at a known concentration in the reaction buffer) and the enzyme concentration in the reaction to obtain *V*_0_/*E*_0_ values. Initial velocities were then fit to the Michaelis–Menten equation to determine the steady-state kinetic parameters for each enzyme variant. Point mutants were generated using the Quikchange method with primer pairs P03/P24.

### Stopped-flow experiments

All stopped-flow experiments were completed in 40 mM HEPES (pH 7.4), 100 mM NaCl and 10% glycerol at 22 °C using a TgK Scientific SF-61DX2 KinetAsyst stopped-flow instrument. A sample consisting of 35 µM NicA2 variant (flavin concentration) was placed in a glass tonometer and made anaerobic by cycling between argon and vacuum^47^. For experiments monitoring the reaction with O_2_ or CycN, NicA2 was stoichiometrically reduced by titration with dithionite in buffer in a gas-tight syringe until the visible absorbance spectrum reached that of reduced flavin. After flavin reduction, NMM or myosmine (kept in a tonometer side arm during anaerobic flavin reduction) was mixed with the reduced enzyme to a concentration of 2 mM ligand. The tonometer was then loaded on the stopped-flow instrument and mixed with buffer containing various O_2_ concentrations (prepared by sparging buffer with various N_2_/O_2_ ratios made using a gas blender) or anaerobic CycN (prepared in a tonometer), and the reaction was monitored using the instrument’s multiwavelength charge-coupled-device detector. For experiments monitoring the reaction with nicotine, oxidized anaerobic NicA2 was mixed with various concentrations of nicotine (made anaerobic by sparging with argon), and the reaction was monitored using the instrument’s single-wavelength photomultiplier tube detector at 450 nm.

Stopped-flow data were analyzed using KaleidaGraph. Kinetic traces at 450 nm for the reaction with O_2_ in the absence of ligand and the reaction of wild-type NicA2–NMM with O_2_ were fit to a single exponential function (Eq. 1) to determine the observed rate constant (*k*_obs_) values for each O_2_ concentration. Kinetic traces for all other experiments were fit to a sum of two exponentials (Eq. 2) to determine *k*_obs_ values for the first and second kinetic phases. In Eqs. 1 and 2, Δ*A* is the kinetic amplitude for each phase, *k*_obs_ is the apparent first-order rate constant, and *A*_∞_ is the absorbance at the end of the reaction.

1 $$Y=\Delta A{e}^{-{k}_{{{\mathrm{obs}}}}t}+{A}_{{\rm{\infty }}}$$

2 $$Y={\Delta A}_{1}{e}^{-{k}_{{{\mathrm{obs}}}1}t}+{{\Delta A}_{2}{e}^{-{k}_{{{\mathrm{obs}}}2}t}+A}_{{{\infty }}}$$

Plots of *k*_obs_ against O_2_ concentration for the flavin oxidation event were fit to a line, with the slope providing the second-order rate constant for the reaction between reduced enzyme and O_2_ (${k}_{{{\mathrm{ox}}}}^{{{\mathrm{O}}}_{2}}$). Plots of *k*_obs_ against nicotine concentration were invariant for NicA2 v320. However, the plot of *k*_obs1_ versus nicotine concentration for wild-type and v321 NicA2 showed a hyperbolic dependence and was therefore fit to Eq. 3 to determine the rate constant for flavin reduction (*k*_red_) for this reaction phase.

3 $${k}_{{{\mathrm{obs}}}}=\frac{{k}_{{{\mathrm{red}}}}\left[S\right]}{{K}_{{\mathrm{d}}}+\left[S\right]}$$

### NMM binding to reduced NicA2

Samples of 40 µM wild-type NicA2 or NicA2 v320 were placed in an anaerobic cuvette and made anaerobic by cycling with vacuum and argon^47^. NicA2’s flavin was reduced by titration with a dithionite solution in a gas-tight syringe, after which the syringe was replaced with a gas-tight syringe containing 2 mM NMM (made anaerobic by sparging with argon). The NMM was titrated into reduced NicA2, and absorbance scans were taken after each addition to monitor the development of charge transfer absorbance after NMM binding. The data for the change in absorbance at 510 nm (Δ*A*_510_) were plotted against NMM concentration and fit to Eq. 4 using Kaleidagraph to estimate the binding affinity. In Eq. 4, Δ*ε*_max_ is the maximum change in extinction coefficient, *E*_0_ is the initial enzyme concentration, and *L*_0_ is the ligand concentration.

4 $$\Delta A_{{510}}=\Delta {\varepsilon }_{\max }\left[\frac{{E}_{0}+{L}_{0}+{K}_{{\mathrm{d}}}-\sqrt{{\big({E}_{0}+{L}_{0}+{K}_{{\mathrm{d}}}\big)}^{2}-4{E}_{0}{L}_{0}}}{2}\right]$$

### NicA2 crystallization and structure determination

Purified NicA2 variants and wild-type NicA2 were prepared in PBS. Initial screening for crystallization conditions was performed using the National High-Throughput Crystallization Center at the Hauptman–Woodward Institute^48^. Crystals for NicA2 v321 were obtained using the hanging-drop vapor diffusion method. Protein solutions from 2.5 to 10 mg ml^−1^ were combined 1:1 with a reservoir solution. NicA2 v321 without added NMM was combined with 100 mM sodium citrate tribasic dihydrate (pH 5.0) and 18% PEG 6000 and set to incubate at 4 °C. For the NMM-bound crystals, NicA2 v321 with NMM supplemented to 20 mM was combined with 80 mM sodium citrate tribasic dihydrate (pH 5.0) and 18% PEG 6000 and incubated at 4 °C. Crystals were collected using the same buffer as the reservoir solution with ethylene glycol added to 25% (vol/vol) as a cryoprotectant before being flash-frozen in liquid nitrogen. For the NMM-bound wild-type crystals, 15 mg ml^−1^ wild-type NicA2 was supplemented with 2.3 mM NMM and combined 1:1 with 0.2 M ammonium citrate tribasic (pH 7.0) and 20% PEG 3350 and set to incubate at 4 °C using the hanging-drop method. Crystals were collected using the same buffer with 15% PEG 3350 added as a cryoprotectant. Crystal diffraction data were collected at the Life Sciences Collaborative Access Team beamline 21-ID-G at the Advanced Photon Source, Argonne National Laboratory. Data integration and scaling were performed with iMosflm^49^ and AIMLESS, respectively. The space group for NicA2 v321 apo crystal and NMM-bound crystal was determined to be *P*4_3_, and the space group for the NMM-bound wild-type NicA2 was determined to be *P*2_1_2_1_2_1_. An asymmetric unit for all three crystals contains two molecules, forming a physiological dimer. All structures were solved by molecular replacement using PHENIX Phaser-MR^50^ with the wild-type NicA2 apo structure (PDB ID 7C4A) as an initial searching model. Multiple rounds of structural refinement and manual model building were performed in the PHENIX Refine program^51^ and Coot^52^. Crystallographic data and refinement statistics are provided in Supplementary Table 3.

### Hydrogen–deuterium exchange mass spectrometry

An automated HDX liquid handling system (LEAP Technologies) coupled with an Acquity M-Class LC/HDX manager (Waters) was used for all experiments. Samples comprised protein (at a concentration of 8 µM with or without 5 mM NMM) in 10 mM potassium phosphate (pH 7.6). HDX was initiated by adding 95 μl of deuterated buffer (10 mM potassium phosphate, pD 7.6) to 5 μl of protein-containing solution. The resultant mixture was incubated at 4 °C for 0.5, 2 or 5 min. Three replicate measurements were performed for each time point and condition. Quenching of the HDX reaction was achieved by mixing 50 μl of the labeling reaction with 100 μl of 10 mM potassium phosphate and 0.05% *n*-dodecyl β-d-maltoside (pH 2.2). Proteolysis was performed by taking 50 μl of the quenched sample and flowing it through an immobilized pepsin column (Waters; 20 °C). Peptides were then trapped (Acquity UPLC BEH C18 VanGuard precolumn, 1.7 μm, 2.1 mm × 5 mm; Waters) for 3 min. The peptides were separated by gradient elution of 0–40% (vol/vol) acetonitrile (0.1% (vol/vol) formic acid) in water (0.3% (vol/vol) formic acid) over 7 min at 40 μl min^−1^ using a C18 column (75 μm × 150 mm; Waters). The eluate was infused into a Synapt G2Si mass spectrometer (Waters) operating in HDMS^E^ mode, with dynamic range extension enabled. Peptides were separated by ion mobility before collision-induced dissociation fragmentation in the transfer cell. Peptides were identified using PLGS (v3.0.2), and isotopic envelopes of the peptides were interrogated using DynamX (v3.0.0) software (Waters). The following PLGS search parameters were used: peptide and fragment tolerances = automatic, min fragment ion matches = 1, digest reagent = non-specific, and false discovery rate = 4. The following DynamX parameters were used: minimum intensity = 1,000, minimum products per amino acid = 0.3, max sequence length = 25, max ppm error = 5, and file threshold = 3. Deuteros 2.0 was used to identify peptides with statistically significant increases/decreases in deuterium uptake and to prepare Woods plots^53^. A summary of the HDX-MS data, as recommended by reported guidelines, is shown in Supplementary Table 2.

### Circular dichroism of NicA2 variants

Samples of 10 μM NicA2 protein (flavin concentration) were prepared in 10 mM potassium phosphate (pH 7.4) and 2.5% (wt/vol) glycerol for circular dichroism experiments. Solutions were added to 1-mm quartz cuvettes (Jasco, 0556). Five scans performed at room temperature were averaged from 260 to 195 nm using a Jasco J-1500 circular dichroism spectrophotometer.

### ThermoFAD melting assay

The ThermoFAD assay was performed as previously established^20^. Briefly, the intrinsic fluorescence of FAD is quenched after being bound by protein. As the temperature of the protein is raised and it begins to unfold, the released FAD is once again fluorescent. This unfolding signal is monitored by a quantitative PCR machine. Twenty microliters of 10 μM NicA2 enzymes was pipetted to the bottom of a quantitative PCR tray and monitored for fluorescence as the temperature was ramped from 15 to 100 °C at a rate of 0.5 °C s^–1^ with a 30-s hold time per step in a QuantStudio 3 Real-Time PCR System.

### Preparation of 4-trifluoromethyl-l-phenylalanine-labeled NicA2 for nuclear magnetic resonance

^19^F-labeled proteins were prepared based on protocols described in previous studies^54^. Quikchange mutagenesis was used to place the amber stop codon (TAG) into desired locations within the *nicA2* gene using primers P25–P28. These genes were then restriction cloned into pET21 at NdeI and XhoI sites with a C-terminal His tag. As such, any unlabeled proteins prematurely truncated at the amber stop codon will pass in the flow-through during a His tag purification step. Overnight culture of *E. coli* BL21 containing the plasmids pDule-tfmF and pET21-*nicA2* was subcultured into 1 liter of PEM supplemented with 1 mM tfmF (Thermo Fisher). Antibiotic selection was maintained using 100 μg ml^–1^ spectinomycin and 200 μg ml^–1^ ampicillin. Cultures were grown to an OD_600_ of 0.8, transferred to a 20 °C shaking incubator and induced overnight with 0.1 mM IPTG. The next day, cells were spun down, and pellets were stored at −20 °C before use. Cell pellets were suspended in 50 ml of lysis buffer supplemented with phenylmethylsulfonyl fluoride, lysozyme and benzonase nuclease and sonicated until cleared. Lysates were spun at 25,000*g* for 30 min and filtered before loading onto a 5-ml HisTrap column (Cytiva) preequilibrated in lysis buffer. The HisTrap column was washed with five column volumes of lysis buffer and eluted in lysis buffer with 300 mM imidazole. This eluate was diluted into 25 mM Tris (pH 8.0) before loading onto a Q-column preequilibrated in the same buffer. The column was then washed with 25 ml of the equilibration buffer before elution in a salt gradient. Purified proteins were dialyzed into 40 mM HEPES (pH 7.4), 100 mM NaCl and 10% (wt/vol) glycerol, snap-frozen and stored at −80 °C before use.

### Nuclear magnetic resonance sample preparation

Purified NicA2 at a concentration of 100–300 μM in 40 mM HEPES (pH 7.4), 100 mM NaCl and 10% (wt/vol) glycerol buffer was used in all NMR reactions unless otherwise specified. A Wilmad internal reference tube containing the same buffer with an added 10% heavy water and 1 mM trifluoroacetic acid (TFA) was used to reference the apo protein spectra. NMM and TEMPOL titration experiments included 10% heavy water with the sample and were instead referenced to free tfmF at a concentration of 5 μM. NMM titrations used 100 μM NicA2 proteins and were referenced to 5 μM TFA in the NMR tube containing an added 10% heavy water. TEMPOL was added to an end concentration of 8 mM. Samples with nicotine added to obtain the reduced product-bound form of the enzyme were prepared by combining NicA2 proteins at a concentration of 300 μM with nicotine to 1 mM to fully reduce FAD. The samples were introduced into NMR tubes and capped with a Chemglass rubber septum stopper. Reduction of the protein throughout all NMR experiments was confirmed by monitoring the color of the protein in the tube.

### ^19^F nuclear magnetic resonance spectroscopy

All NMR spectra were acquired at 298 K on a Bruker 600 MHz instrument equipped with a Prodigy (^1^H/^19^F)-X broadband cryoprobe operating at a basic transmitter frequency of 563.6701729 MHz for the ^19^F nucleus. One-dimensional undecoupled ^19^F spectra were recorded with 16,000 data points, a 30-kHz sweep width, an acquisition time of 0.288 s, a relaxation delay of 2 s and a total of 1,000–5,000 transients accumulated per experiment. The ^19^F chemical shifts were referenced to either TFA (set at −75.39 ppm) or tfmF (set at –62.0832 ppm). The spectra were processed in TopSpin 4.1.4 and deconvoluted using the Python-based decon1d fitting program that assumes Lorentzian peak shapes^44^.

### Albumin binding domain fusion proteins

*nicA2* sequences were fused to the nucleotide sequence of ABD035 (ref. ^55^) in a PCR assembly reaction using a primer pair (P29/P30) in which the forward primer encoded the entirety of ABD035 and primed on the N terminus of NicA2 after its signal sequence. This construct was the same architecture as that of an earlier study where ABD–NicA2 was shown to be stable and active^4^. The resulting fusion was cloned into pET28a-His-SUMO via BamHI and XhoI restriction sites. The protein was purified as detailed above for the crystallography and stopped-flow purifications. Endotoxin was removed from the samples using the Toxineraser endotoxin removal kit (Genscript) and confirmed to be lower than 5 EU ml^–1^ with a Toxinsensor chromogenic LAL endotoxin assay kit (Genscript). The samples were prepared in 40 mM HEPES (pH 7.4), 100 mM NaCl and 10% (wt/vol) glycerol and flash-frozen in liquid nitrogen before use.

### Plasma collection to determine nicotine levels in rats injected with NicA2 variants

Adult male and female Wistar rats (*N* = 24 males and 24 females) were subcutaneously implanted with osmotic minipumps (Alzet 2ML2, Braintree Scientific) containing 3.15 mg per kg (body weight) per d nicotine (dose expressed as free base) for 7 d, as in our previous work^4,56^. All animal experiments were conducted in compliance with the University of California, San Diego, Institutional Care and Use Committee (IACUC), and all experimental procedures were approved by the University of California, San Diego, IACUC. Briefly, rats were anesthetized with 1–5% isoflurane in O_2_, and an incision was made in the lumbar region. Minipumps were inserted subcutaneously, the incision was closed with wound clips and skin glue, and minipumps were removed after 7 d. At the time of minipump implantation and every 2 d after, animals were intraperitoneally injected with vehicle (1× PBS; naive and vehicle groups, *n* = 4 males and 4 females per group), 0.1 mg per kg (body weight) wild-type ABD–NicA2 (*n* = 4 males and 4 females), 1 mg per kg (body weight) wild-type ABD–NicA2 (*n* = 4 males and 4 females), 0.1 mg per kg (body weight) ABD–NicA2 v321 (*n* = 4 males and 4 females) or 1 mg per kg (body weight) ABD–NicA2 v321 (*n* = 4 males and 4 females). At 1 and 5 d after minipump implantation, blood was collected from each animal via tail vein puncture and immediately mixed with 1:4 volumes of methanol to quench enzyme activity. Samples were spun at 1,000*g* for 10 min at room temperature, and plasma was extracted and stored at −80 °C until use.

### Determination of plasma nicotine levels by mass spectrometry

Calibrators and quality control material were prepared from standard reference material: nicotine (Sigma-Aldrich). Internal standards (ISs) were prepared from standard reference material: nicotine -d3 (Cayman Chemical). Calibration standards were prepared in rat plasma and were used to generate an external calibration curve using linear regression to plot the peak area ratio versus concentration with 1/× weighting (*r*^2^ ≥ 0.99) over the full analytically reportable range. The analytically reportable range for this assay was 7.8–250 ng ml^−1^ for nicotine. Quantitative determination of nicotine in rat plasma was determined by multiple reaction monitoring (MRM) MS API 4000 LC–MS/MS System-Sciex. MRM was performed in positive electrospray ionization mode using column C18-AR (100 × 2.1 mm; 5-µm ACE). MRM transition ions were monitored for nicotine (quantifier: *m*/*z* 163.0 > 117.0; qualifier: *m*/*z* 163.0 > 130.0; IS: *m*/*z* 166.0 > 132.0).

The analytical quality control materials were prepared at 15 ng ml^−1^ and 150 ng ml^−1^ in mouse plasma. Mobile phase A (MPA) was composed of 0.1% formic acid in water, and mobile phase B (MPB) was composed of methanol. The flow rate was set at 0.500 ml min^−1^ across the entire method, and the initial starting conditions were set at 97% MPA and 3% MPB. At 1.00 min, the composition of MPB increased linearly to 97% across the following 2.50 min. MPB was held constant for 1.50 min before returning to 5% MPB. Equilibration was performed for the final 1.1 min at 5% MPB. Nicotine was extracted from 50 µl of rat plasma spiked with 20 µl of IS. Proteins were precipitated using 180 µl of methanol and vortexed for 30 s before centrifugation at 21,000*g* for 10 min at room temperature. A volume of 20 µl was injected via the autosampler for LC–MRM analysis.

### Statistics and reproducibility

Independent replicates were obtained for all experiments included in the study and are typically presented as mean values or comparisons of means. Measurements were repeated at least two times, and all were reproducible. For rat studies, pilot testing was conducted on a smaller cohort of animals, and results were consistent with data presented in the manuscript.

### Reporting summary

Further information on research design is available in the Nature Portfolio Reporting Summary linked to this article.

## Online content

Any methods, additional references, Nature Portfolio reporting summaries, source data, extended data, supplementary information, acknowledgements, peer review information; details of author contributions and competing interests; and statements of data and code availability are available at 10.1038/s41589-023-01426-y.

### Supplementary information

Supplementary Information Supplementary Tables 1–4 and Figs. 1–7.

Reporting Summary

### Source data

Source Data Fig. 1 Data used to generate plots.

Source Data Fig. 2 Data used to generate plots.

Source Data Fig. 3 Data used to generate plots.

Source Data Fig. 4 Data used to generate plots.

Source Data Fig. 5 Data used to generate plots.

Source Data Extended Data Fig. 1 Data used to generate plots.

Source Data Extended Data Fig. 3 Data used to generate plots.

Source Data Extended Data Fig. 5 Data used to generate plots.

Source Data Extended Data Fig. 10 Data used to generate plots.

## Figures and Tables

**Extended Data Fig. 4:**
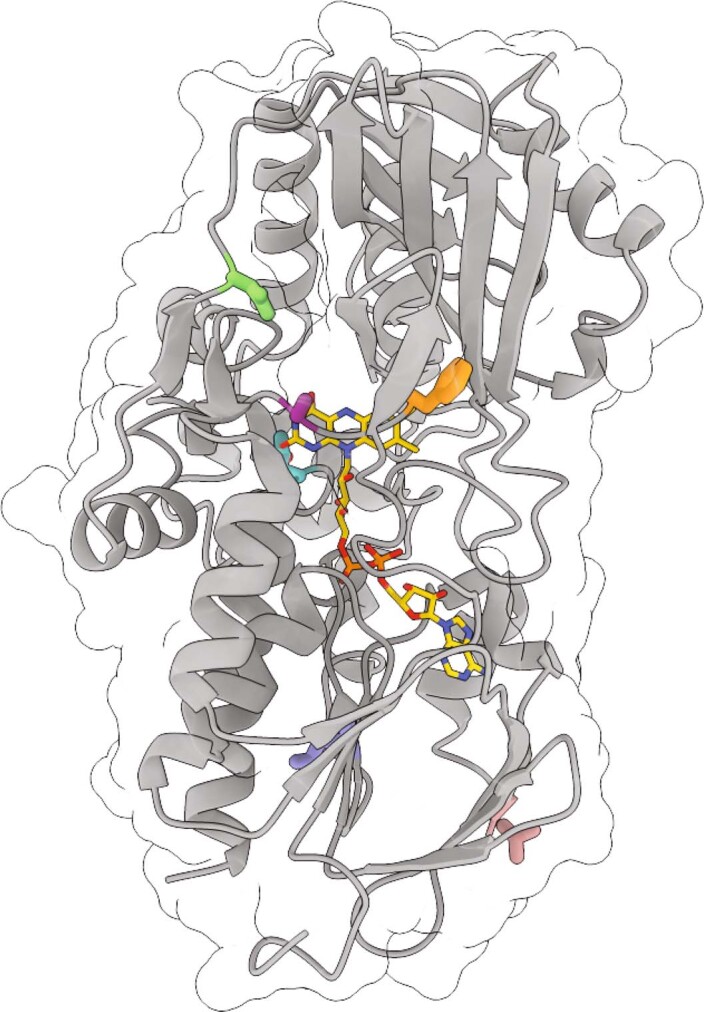
Mutations in NicA2 v320. The crystal structure of wildtype NicA2 is displayed with residues highlighted that are mutated in NicA2 v320. Note F104 in orange, A107 in purple, D130 in green, T319 in salmon, L449 in cornflower blue, and N462 in turquoise.

## Data Availability

Raw data used in the preparation of main and Extended Data figures are deposited in the public data repository Figshare (10.6084/m9.figshare.23635986). Macromolecular structure data are available at the PDB (https://www.wwpdb.org/) with the identifiers listed in this study. The raw HDX-MS data have been deposited to the ProteomeXchange Consortium via the PRIDE partner repository with the dataset identifier PXD037151. Datasets generated during and/or analyzed during the current study are available from the corresponding author on reasonable request. Source data are provided with this paper.
