# Supplementary material for: Directed evolution unlocks oxygen reactivity for a nicotine-degrading flavoenzyme
Source: Nat Chem Biol. 2023 Sep 28;19(11):1406–14. doi: 10.1038/s41589-023-01426-y (PMC10611581; doi:10.1038/s41589-023-01426-y)
Supplement: Supplementary file 1 — Supplementary Tables 1–4 and Figs. 1–7. [file 41589_2023_1426_MOESM1_ESM.pdf]

nature chemical biology

Article

https://doi.org/10.1038/s41589-023-01426-y

Directed evolution unlocks oxygen reactivity for a nicotine-degrading flavoenzyme

In the format provided by the authors and unedited

nature chemical biology

Article

https://doi.org/10.1038/s41589-023-01426-y

Directed evolution unlocks oxygen reactivity for a nicotine-degrading flavoenzyme

In the format provided by the authors and unedited

Directed evolution unlocks oxygen reactivity for a nicotine degrading flavoenzyme Supplementary Information

Supplementary Table 1. Steady-state kinetic data of NicA2 wildtype and variants

NicA2 variant	Encoded mutations	k_{cat} (s^{-1})[a]	K_M (μM)[a]	k_{cat}/K_M ($M^{-1}s^{-1}$)
WT		$0.0066 \pm 2 \times 10^{-4}$	0.114[b]	5.4×10^{4} [b]
6	H368R	0.0837 ± 0.008	ND	ND
15	A107T, D130N, S192N, M265I, T397A	0.156 ± 0.004	1.9 ± 0.6	8.2×10^{4}
16	E249G, H368R	0.166 ± 0.004	1.1 ± 0.4	1.5×10^{5}
177	G145A, S192I, Q359L, Q366R, S379N	0.17 ± 0.01	60 ± 10	2.7×10^{3}
235	K49N, F93L, F104L, V127M, D130S, L132R, F174C, T319I, E454D	0.274 ± 0.004	23 ± 2	1.2×10^{4}
254	A107T, D130G, T267I, N462S	0.32 ± 0.01	15 ± 2	2.1×10^{4}
255	F104L, G317D, H368R, L449V, N462S	0.239 ± 0.004	1.1 ± 0.3	2.2×10^{5}
305	F93L, F104L, V127M, D130S, L132R, S213T, T222S, A281T, L374M, M403I	0.52 ± 0.01	140 ± 10	3.9×10^{3}
320	F104L, A107T, D130S, T319I, L449V, N462S	1.06 ± 0.01	6.3 ± 0.4	1.7×10^{5}
321	F104L, A107T, S146I, G317D, H368R, L449V, N462S	0.57 ± 0.01	0.9 ± 0.3	6.3×10^{5}
416	T67A, F104L, A107T, D130S, K165R, N462S	0.93 ± 0.04	8 ± 2	1.2×10^{5}
417	G50D, F104L, A107T, D130S, F422L, N462H	1.25 ± 0.04	7 ± 1	1.8×10^{5}
426	G50D, F104L, A107T, D130S, T319I, L410M, L449V, N462S	1.04 ± 0.02	7.7 ± 0.9	1.4×10^{5}
427	F104L, T280A, D294Y, T307S, G317D, N363S, H368R, S379N, N462S	0.65 ± 0.02	4.1 ± 0.7	1.6×10^{5}

Values reported with the 95% confidence interval from 3 technical replicates.
[a] The steady-state kinetic assays in this study were performed under ambient conditions, meaning that the k_{cat} and K_M values discussed here represent an *apparent* k_{cat} and K_M for nicotine turnover at an oxygen concentration of roughly 250 μM.
[b] Denotes values reported from Tararina et al.[1]

Supplementary Table 2. HDX Data Summary.

**Data Set**	**Wildtype NicA2**	**NicA2 v321**	**Wildtype NicA2 + NMM**	**NicA2 v321 + NMM**
**HDX reaction details**	10 mM potassium phosphate, pD 7.6, 4 ºC	10 mM potassium phosphate, pD 7.6, 4 ºC	10 mM potassium phosphate, pD 7.6, 4 ºC	10 mM potassium phosphate, pD 7.6, 4 ºC
**HDX time course (min)**	0.5, 2, 5, 10 or 30 min			
**HDX control samples**	Maximally labeled controls were not performed.			
**Back-exchange**	~ 30 %			
**# of Peptides**	105	81	105	81
**Sequence coverage**	91 %	81 %	91 %	81 %
**Average peptide length / Redundancy**	12.47/ 2.98	11.75 / 2.44	12.47/2.98	11.75 / 2.44
**Replicates (biological or technical)**	3 (technical)	3 (technical)	3 (technical)	3 (technical)
**Repeatability**	0.030 (average SD)	0.048 (average SD)	0.043 (average SD)	0.045 (average SD)
**Significant differences in HDX (delta HDX > XD)**	Reference	Hybrid Significance test: 99% CI: 0.27 Da/ p-value <-0.01. A Welch's t-test was used to confirm the significance using a p-value cutoff of 0.01.	Reference	Hybrid Significance test: 99% CI: 0.29 Da/ p-value <-0.01. A Welch's t-test was used to confirm the significance using a p-value cutoff of 0.01.

SD = standard deviation, CI = confidence interval.

Supplementary Table 3. Statistics of X-ray structure determination.

	NicA2 v321-apo	NicA2 v321-NMM	WT-NMM
**Data collection**			
**Space group**	P43	P43	$P2_12_12_1$
**Cell dimensions**			
a, b, c **(Å)**	85.5, 85.5, 122.4	86.3, 86.3, 122.5	75.1, 86.7, 152.1
**α, β, γ (°)**	90.0, 90.0, 90.0	90.0, 90.0, 90.0	90.0, 90.0, 90.0
**Resolution (Å)**	42.76-2.10 (2.16-2.10)	32.64-1.90 (1.94-1.90)	57.18-2.50 (2.60-2.50)
**R_{merge} (%)**	7.6 (47.6)	8.0 (46.5)	10.3 (31.2)
**R_{meas} (%)**	8.2 (51.1)	8.6 (49.9)	11.1 (33.6)
**$I/\sigma(I)$**	17.6 (4.3)	15.9 (4.2)	11.7 (5.2)
**$CC_{1/2}$**	0.907 (0.818)	0.823 (0.796)	0.894 (0.879)
**Redundancy**	7.6 (7.5)	7.6 (7.5)	7.0 (7.2)
**Completeness (%)**	100 (100)	100 (100)	97.2 (89.2)
**Total/unique reflections**	388225/51280 (31529/4187)	536368/70427 (34209/4531)	239376/34120 (24554/3430)
**Refinement**			
**R_{work}, R_{free} (%)**	15.1, 20.0	14.0, 17.7	16.4, 21.2
**No. atoms**	7279	7499	7149
**Protein**	6661	6704	6610
**Ligand**	172	192	275
**Water**	446	603	264
**Rotamer outliers (%)**	0.59	0.15	0.6
**Clashscore**	4.2	3.57	2.72
**RMSD**			
**Bond lengths (Å)**	0.012	0.016	0.004
**Bond angles (°)**	1.05	1.19	0.69
**Average B factors (Å^2)**	29.18	21.77	34.90
**Wilson B-factor (Å^2)**	26.80	20.07	31.88
**Ramachandran statistics**			
**Favored regions (%)**	97.56	98.49	97.33
**Allowed regions (%)**	2.44	1.51	2.44
**Outliers (%)**	0.00	0.00	0.23

Statistics for the highest-resolution shell are shown in parentheses.

Supplementary Table 4. Primers used in this study.

Primer name	Primer sequence (5' to 3')
**P01**	ACCCAGAAGACCAACCGTG
**P02**	TTAGCTCAGCAGTTGTTTCACTTCACG
**P03**	CGGGTCAAGAGATCGAGTTAGGCGGTGCGT
**P04**	ACGCACCGCCTAACTCGATCTCTTGACCCG
**P05**	GATCGAGTTCGGCGGTACGTGGGTTCATTGG
**P06**	CCAATGAACCCACGTACCGCCGAACTCGATC
**P07**	CGGCCTGGGTGTGGTTGAAAGTCCGCTGACCAA
**P08**	TTGGTCAGCGGACTTTCAACCACACCCAGGCCG
**P09**	CTCGTCGCTATAATCGCGGGTTTGCACCCAGTT
**P10**	AACTGGGTGCAAACCCGCGATTATAGCGACGAG
**P11**	GGCTGGCACGCGAGCATTGATGGTGCG
**P12**	CGCACCATCAATGCTCGCGTGCCAGCC
**P13**	GTCAAGAGATCGAGTTCGGCGGTACGTGG
**P14**	CCACGTACCGCCGAACTCGATCTCTTGAC
**P15**	ATCGAGCTCGGCGGTGCGTGGGTTCATTG
**P16**	CAATGAACCCACGCACCGCCGAGCTCGAT
**P17**	CGGCCTGGGTGTGGTTGAAGATCCGCTGACCAA
**P18**	TTGGTCAGCGGATCTTCAACCACACCCAGGCCG
**P19**	TACAAACACATCGGCTTCACCCCGGCGCTG
**P20**	CAGCGCCGGGGTGAAGCCGATGTGTTTGTA
**P21**	CGGAGGGTCGTATCTTGTTTGCTGGTGCG
**P22**	CGCACCAGCAAACAAGATACGACCCTCCG
**P23**	CGGCTGGCACGCGAATATTGATGGTGCTG
**P24**	CAGCACCATCAATATTCGCGTGCCAGCCG
**P25**	GTGCCGCTGAACACCTAGAAACACATCGGCT
**P26**	AGCCGATGTGTTTCTAGGTGTTCAGCGGCAC
**P27**	GCAAGGGTGCGAAACTGTAGGTTCACGTGAAACAG
**P28**	CTGTTTCACGTGAACCTACAGTTTCGCACCCTTGC
**P29**	ATATAGGATCCATGGATGCCAACAGCCTGGCTGAAGCAAAAGTGTTGGCCAATC GCGAGCTGGATAAATATGGCGTGAGCGACTTCTATAAACGCTTGATCAATAAAG CTAAGACCGTGGAAGGCGTTGAAGCTCTGAAACTTCATATTTTGGCTGCACTGC CAAGCGGTGGCTTCGATTACGACGTGGTTGTG
**P30**	GCGCGCCTCGAGTTAGCTCAGCAGTTG

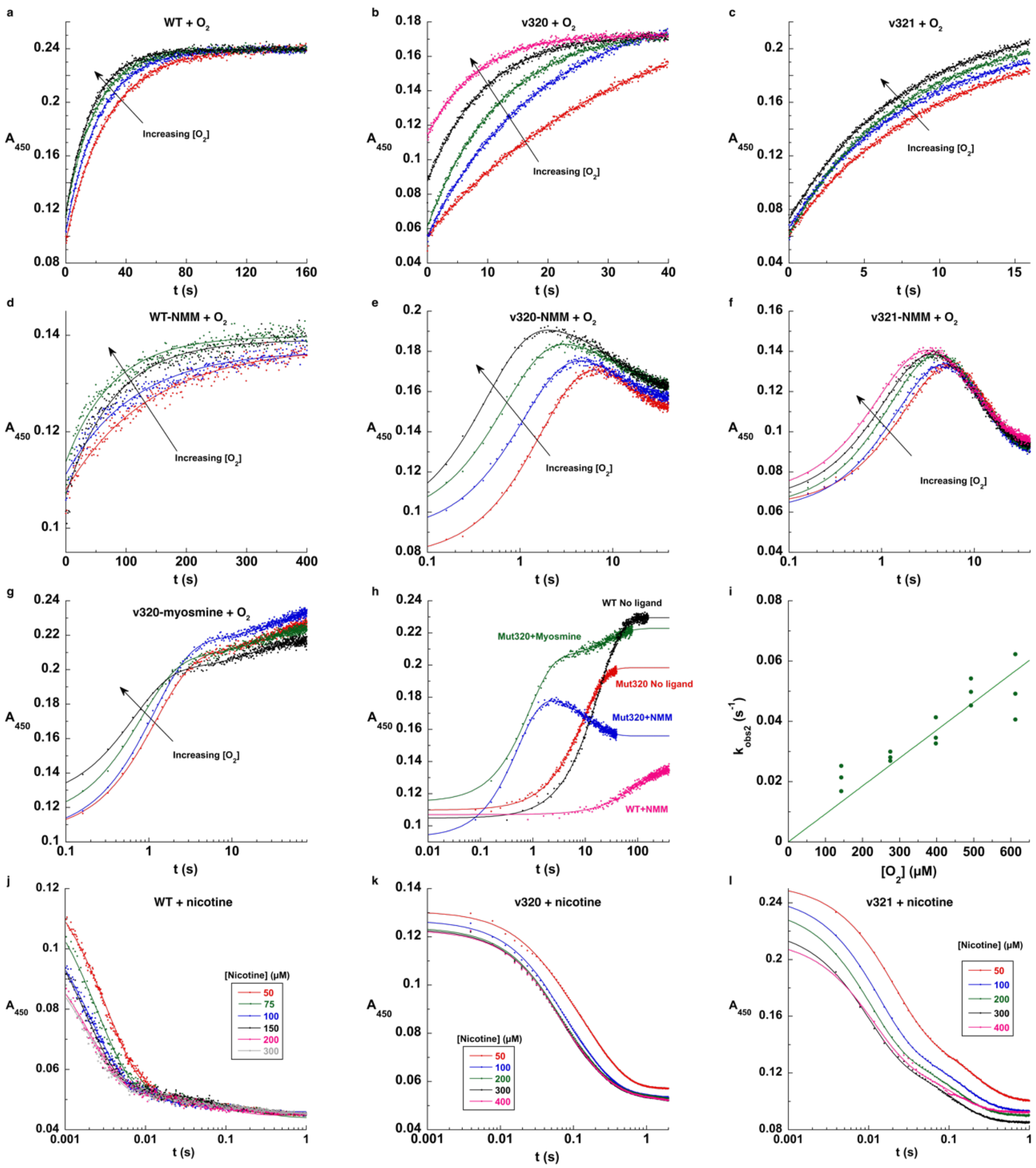

Supplementary Figure 1. Raw traces of stopped flow reactions. a, The reaction of reduced NicA2 wildtype with oxygen. **b,** The reaction of reduced NicA2 v320 with oxygen. **c,** The reaction of reduced NicA2 v321 with oxygen. **d,** The reaction of reduced NicA2 wildtype in the presence of 1 mM NMM with oxygen. **e,** The reaction of reduced NicA2 v320 in the presence of 1 mM NMM with oxygen. **f,** The reaction of reduced NicA2 v321 in the presence of 1 mM NMM with oxygen. **g,** The reaction of reduced NicA2 v320 in the presence of 1 mM myosmine with oxygen. **h,** Overlays of NicA2 under various conditions reacting with oxygen at 450 µM. **i,** Plot of the k_{obs2} values for the second phase of oxidation for reduced NicA2 v320 in the presence of myosmine reacting with oxygen. The slope of the fitted line (93 ± 45 $M^{-1}s^{-1}$) is comparable to that of ligand-free NicA2 v320 (**Table 1** of main text). **j,** The reaction of NicA2 wildtype with nicotine. **k,** The reaction of NicA2 v320 with nicotine. **l,** The reaction of NicA2 v321 with nicotine.

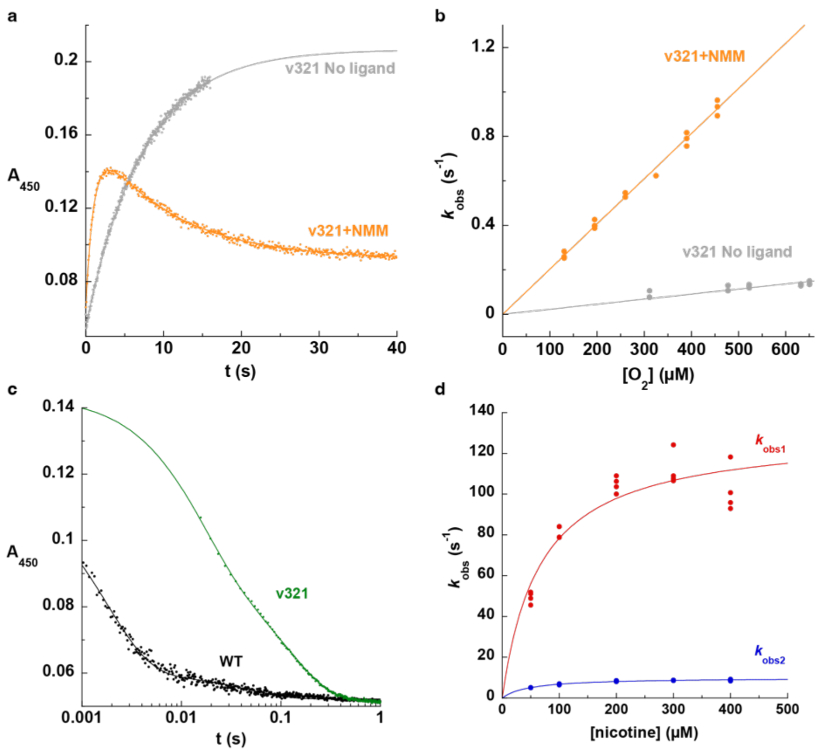

Supplementary Figure 2. Stopped flow reactions of NicA2 v321. a, Example traces for the stopped-flow reaction of reduced NicA2 v321 with O_2 under the described conditions. The decrease in absorbance that occurs after flavin oxidation for the NMM-bound trace is caused by an interaction between NMM and NicA2 containing oxidized FAD[2]. **b,** k_{obs} values for the first phase of oxidation are plotted against the concentration of O_2 and fit to a line. The slopes of these lines define the bimolecular rate constants for oxidation by O_2 and are presented for the various conditions in **Table 1** of the main text. **c,** Example traces for the stopped-flow reaction of oxidized NicA2 WT and v321 with nicotine. Note the logarithmic x-axis. **d,** k_{obs} values for the two phases observed in the reaction of NicA2 v321 with nicotine are plotted against the concentration of nicotine used in each experiment and then fit to a hyperbola. The resulting rate constants for reduction are presented in **Table 1** of the main text.

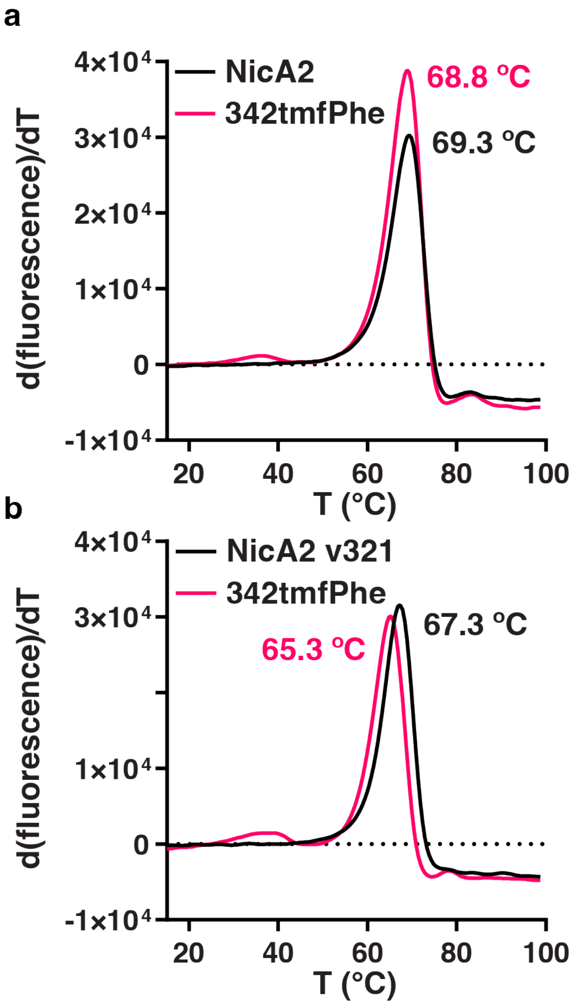

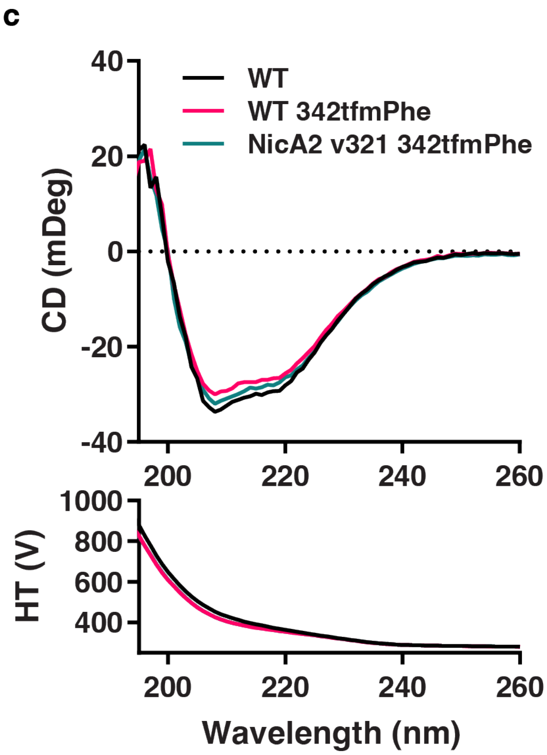

d

	k_{cat} (s^{-1})
WT	0.0066 ± 2 × 10^{-4}
WT 342tmfPhe	0.032 ± 0.002
v321	0.58 ± 0.01
v321 342tmfPhe	0.476 ± 0.008

Supplementary Figure 3. Qualities of NicA2 ^{19}F containing variants. a, NicA2 wildtype and NicA2 wildtype with tfmPhe substituted at position 342 have a similar melting temperature. Traces represent an average of 3 replicates. **b,** NicA2 v321 and NicA2 v321 with tfmPhe substituted at position 342 have a slightly different, but still similar melting temperature. Traces represent an average of 3 replicates. **c,** Circular dichroism spectra of wildtype and the two ^{19}F containing constructs maintain secondary structure. **d,** Steady-state kinetic data for the reaction of NMR variants with nicotine. Values are reported with 95% confidence intervals.

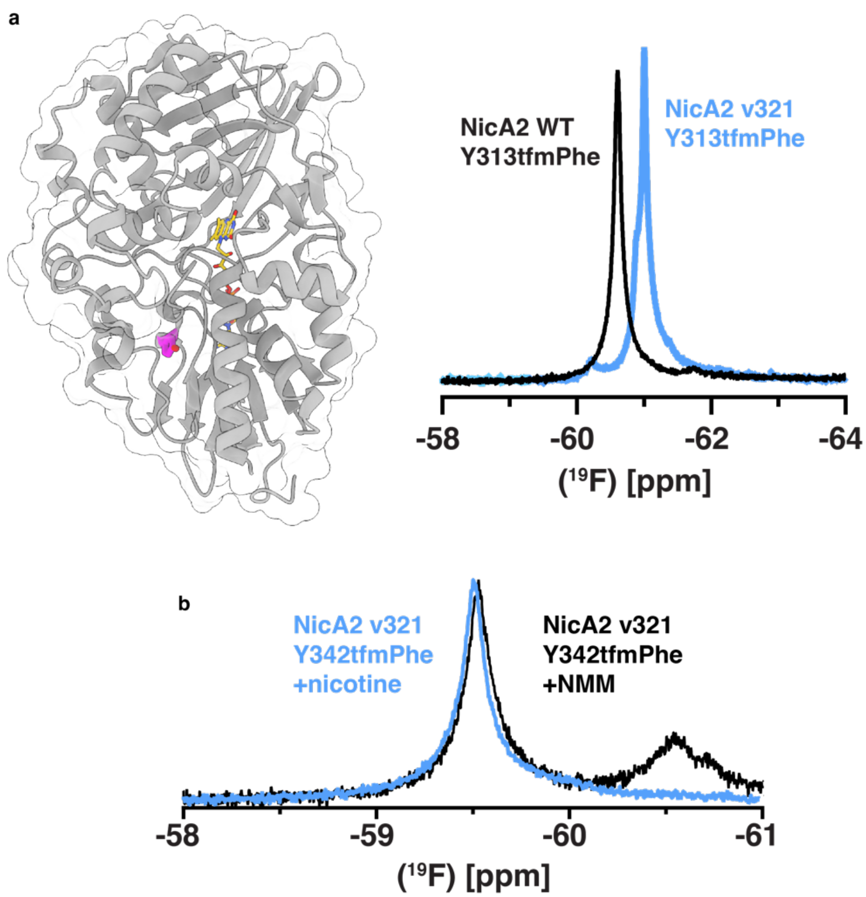

Supplementary Figure 4. A tmfPhe substitution far from the tunnel region in NicA2 does not display conformational heterogeneity. a, Y313 (magenta) was mutated to the amber stop codon, and tfmPhe incorporated into this position to serve as a probe for ^{19}F NMR. This position was chosen because it is conservative substitution distant from the tunnel region and buried in the core of the flavin-binding domain. It should therefore be an independent readout about global protein dynamics that is not influenced by local tunnel dynamics. Displayed in the right panel are the ^{19}F NMR spectra of the Y313tfmPhe substitution for wildtype and variant NicA2s, which are similar. **b,** Nicotine was titrated into NicA2 v321 Y342tmfPhe to achieve the reduced, NMM-bound state. NMM was also titrated into the oxidized enzyme. These major species of these spectra overlay closely, indicating that NMM-bound oxidized and reduced states are conformationally indistinguishable.

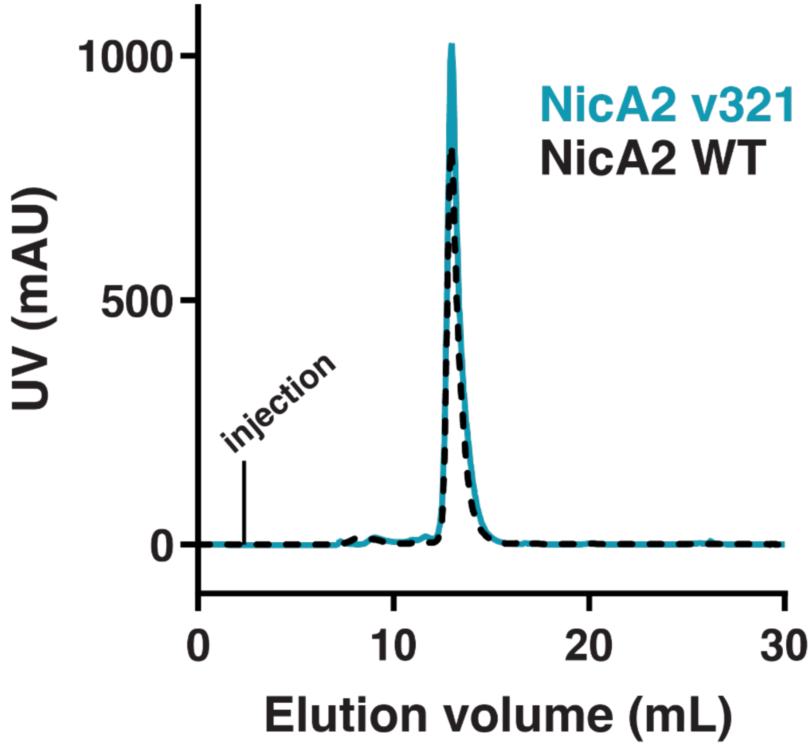

Supplementary Figure 5. NicA2 wildtype and v321 have a similar elution profile in size exclusion chromatography. NicA2 enzymes were prepared at NMR concentrations (100 µM). 200 µL of each enzyme were injected onto a Superdex 200 10/300 GL column equilibrated in 40 mM HEPES pH 7.4, 100 mM NaCl, 10% (w/v) glycerol. An Äkta-FPLC system was used to maintain a flowrate of 0.2 mL min^{-1}, and absorbance of the sample monitored at 280 nm. The elution profile of NicA2 wildtype is displayed as a dashed black line, v321 is a solid cyan line.

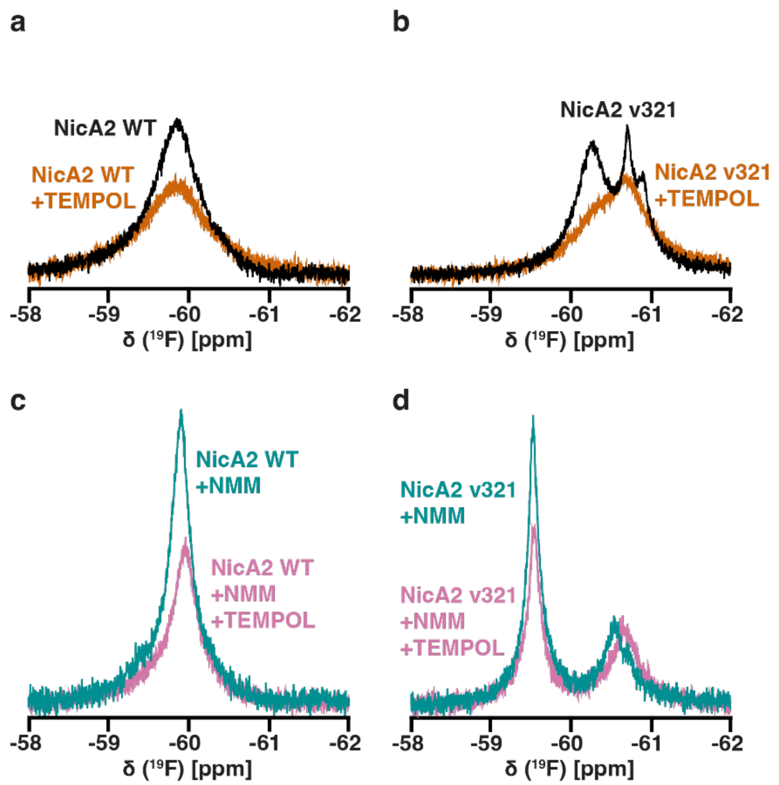

Supplementary Figure 6. NicA2 enzymes upon addition of TEMPOL. a, Scans were taken before and after TEMPOL was added to 8 mM end concentration in samples of NicA2 Y342tmfF wildtype or **b,** v321. **c,** The same experiment was repeated with 5 mM NMM included in samples of NicA2 wildtype and **d,** v321.

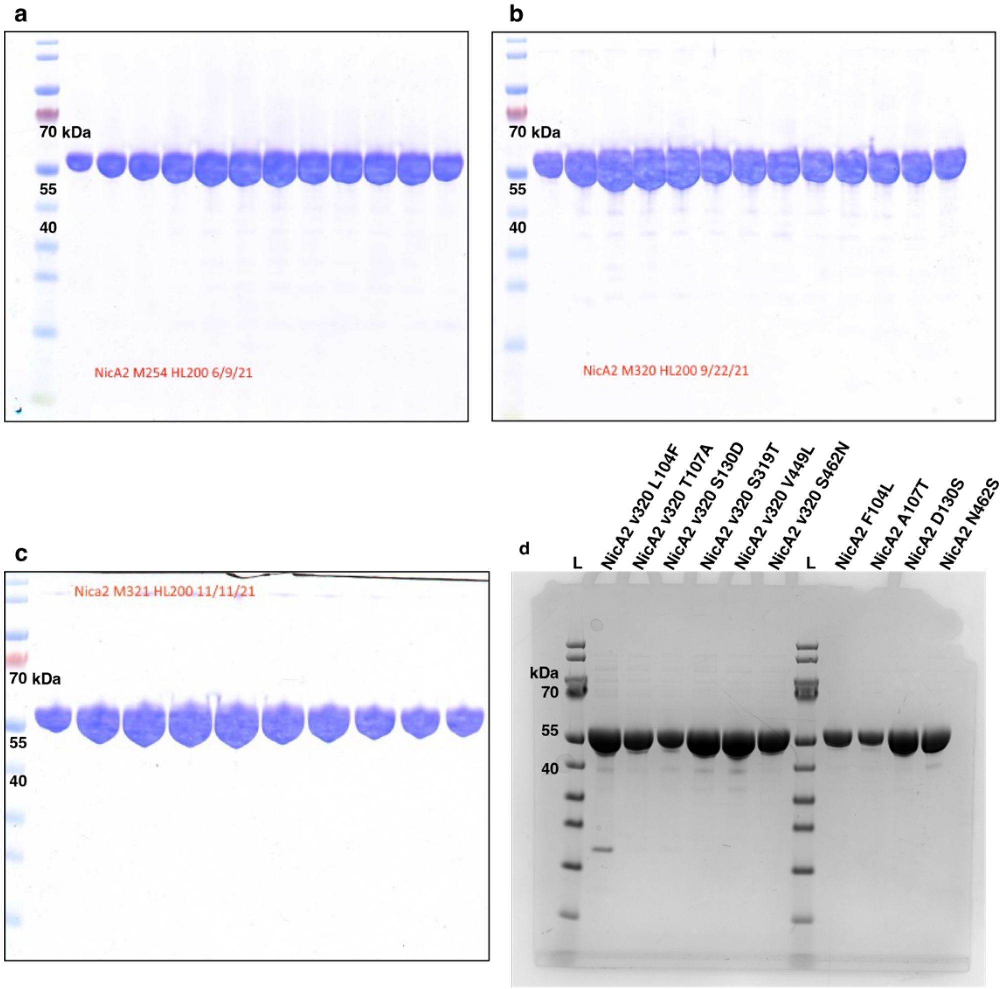

Supplementary Figure 7. SDS-PAGE showing purity of NicA2 variants. a, Fractions collected after size exclusion of NicA2 v254, all fractions were collected and combined. **b,** Fractions collected after size exclusion of NicA2 v320. **c,** Fractions collected after size exclusion of NicA2 v321. **d,** Purity of various NicA2 variants used for steady-state analyses in this study. All proteins were checked for purity using gel electrophoresis before use.

References

1. Tararina, M. A. *et al.* Crystallography coupled with kinetic analysis provides mechanistic underpinnings of a nicotine-degrading enzyme. *Biochemistry* **57**, 3741–3751 (2018).
2. Dulchavsky, M., Clark, C. T., Bardwell, J. C. A. & Stull, F. A cytochrome c is the natural electron acceptor for nicotine oxidoreductase. *Nat. Chem. Biol.* (2021) doi:10.1038/s41589-020-00712-3.
