# Supplementary material for: Directed evolution unlocks oxygen reactivity for a nicotine-degrading flavoenzyme
Source: Nat Chem Biol. 2023 Sep 28;19(11):1406–14. doi: 10.1038/s41589-023-01426-y (PMC10611581; doi:10.1038/s41589-023-01426-y)
Supplement: Supplementary file 2 — Reporting Summary [file 41589_2023_1426_MOESM2_ESM.pdf]

nature portfolio

Corresponding author(s): James Bardwell

Last updated by author(s): Jul 11, 2023

Reporting Summary

Nature Portfolio wishes to improve the reproducibility of the work that we publish. This form provides structure for consistency and transparency in reporting. For further information on Nature Portfolio policies, see our Editorial Policies and the Editorial Policy Checklist.

Statistics

For all statistical analyses, confirm that the following items are present in the figure legend, table legend, main text, or Methods section.

n/a	Confirmed	
☐	☒	The exact sample size (*n*) for each experimental group/condition, given as a discrete number and unit of measurement
☐	☒	A statement on whether measurements were taken from distinct samples or whether the same sample was measured repeatedly
☐	☒	The statistical test(s) used AND whether they are one- or two-sided<br>*Only common tests should be described solely by name; describe more complex techniques in the Methods section.*
☒	☐	A description of all covariates tested
☐	☒	A description of any assumptions or corrections, such as tests of normality and adjustment for multiple comparisons
☐	☒	A full description of the statistical parameters including central tendency (e.g. means) or other basic estimates (e.g. regression coefficient) AND variation (e.g. standard deviation) or associated estimates of uncertainty (e.g. confidence intervals)
☐	☒	For null hypothesis testing, the test statistic (e.g. *F*, *t*, *r*) with confidence intervals, effect sizes, degrees of freedom and *P* value noted<br>*Give P values as exact values whenever suitable.*
☒	☐	For Bayesian analysis, information on the choice of priors and Markov chain Monte Carlo settings
☒	☐	For hierarchical and complex designs, identification of the appropriate level for tests and full reporting of outcomes
☒	☐	Estimates of effect sizes (e.g. Cohen's *d*, Pearson's *r*), indicating how they were calculated

Our web collection on statistics for biologists contains articles on many of the points above.

Software and code

Policy information about availability of computer code

Data collection	Bruker TopSpin 4.1.4, Kinetic Studio 5.10.6, UV Probe 2.70, HDX-MS data were acquired using software supplied by the instrument vendor (Masslynx v4.2, Waters).
Data analysis	TopSpin 4.1.4, Excel (Microsoft Office 365), GraphPad Prism 9, decon1d program (https://github.com/hughests/deconld), KaleidaGraph 4.5.4, Coot 0.9.6. HDX-MS data were analyzed using PLGS (v3.0.2) and DynamX (v3.0.0) (Waters). Peptides with statistically significant differences in deuterium incorporation were identified using the software Deuteros 2.4.2.

For manuscripts utilizing custom algorithms or software that are central to the research but not yet described in published literature, software must be made available to editors and reviewers. We strongly encourage code deposition in a community repository (e.g. GitHub). See the Nature Portfolio guidelines for submitting code & software for further information.

Data

Policy information about availability of data

All manuscripts must include a data availability statement. This statement should provide the following information, where applicable:

- Accession codes, unique identifiers, or web links for publicly available datasets
- A description of any restrictions on data availability
- For clinical datasets or third party data, please ensure that the statement adheres to our policy

Raw data used in the preparation of main and extended data figures are deposited in the public data repository Figshare (https://doi.org/10.6084/

m9.figshare.23635986). Macromolecular structure data is available at the protein data bank (pdb.org) with the identifiers listed in this study. The raw HDX-MS data have been deposited to the ProteomeXchange Consortium via the PRIDE partner repository with the dataset identifier: PXD037151. Other datasets generated during and/or analyzed during the current study are available from the corresponding author on reasonable request.

Research involving human participants, their data, or biological material

Policy information about studies with human participants or human data. See also policy information about sex, gender (identity/presentation), and sexual orientation and race, ethnicity and racism.

Reporting on sex and gender	Not applicable
Reporting on race, ethnicity, or other socially relevant groupings	Not applicable
Population characteristics	Not applicable
Recruitment	Not applicable
Ethics oversight	Not applicable

Note that full information on the approval of the study protocol must also be provided in the manuscript.

Field-specific reporting

Please select the one below that is the best fit for your research. If you are not sure, read the appropriate sections before making your selection.

☒ Life sciences ☐ Behavioural & social sciences ☐ Ecological, evolutionary & environmental sciences

For a reference copy of the document with all sections, see nature.com/documents/nr-reporting-summary-flat.pdf

Life sciences study design

All studies must disclose on these points even when the disclosure is negative.

Sample size	Sample sizes for molecular biology and biochemistry experiments were chosen based on prior literature and accepted standards in the field (doi:10.1021/acs.biochem.0c00855). For the rat studies, sample sizes were chosen based on power analysis to achieve a = 0.05, B = 0.8, and effect sized= 0.5.
Data exclusions	All raw data for the nicotine levels in rats was run through the Grubb's outlier test to identify outliers with an alpha = 0.05. This resulted 3 out of 96 total values being identified as significant outliers and excluded.
Replication	Independent replicates were obtained for all experiments included in the study and are typically presented as mean values or comparisons of means. Measurements were repeated at least two times and all were reproducible. For rat studies, pilot testing was conducted on a smaller cohort of animals (data not shown) and results were consistent with data presented in the manuscript.
Randomization	Allocation was not randomized. There are no relevant covariates in this study and reported values are measured using instruments/techniques that are not subject to interpreter bias. For the rat studies, male and female rats were randomly assigned to treatment groups upon arrival, such that each treatment group had an equal number of male and female rats.
Blinding	For the rat studies, experimental groups were blinded to the experimenter. For the other studies, no blinding was used, the instruments/techniques that were used to gather data were not thought to be subject to interpreter bias

Reporting for specific materials, systems and methods

We require information from authors about some types of materials, experimental systems and methods used in many studies. Here, indicate whether each material, system or method listed is relevant to your study. If you are not sure if a list item applies to your research, read the appropriate section before selecting a response.

Materials & experimental systems

n/a	Involved in the study
☒	☐ Antibodies
☒	☐ Eukaryotic cell lines
☒	☐ Palaeontology and archaeology
☐	☒ Animals and other organisms
☒	☐ Clinical data
☒	☐ Dual use research of concern
☒	☐ Plants

Methods

n/a	Involved in the study
☒	☐ ChIP-seq
☒	☐ Flow cytometry
☒	☐ MRI-based neuroimaging

Animals and other research organisms

Policy information about studies involving animals; ARRIVE guidelines recommended for reporting animal research, and Sex and Gender in Research

Laboratory animals	Male and female Wistar rats, 8-10 weeks at start of study
Wild animals	This study did not involve wild animals
Reporting on sex	Details are listed throughout Methods as part of experimental group descriptions (N = 24M + 24F, etc), as well as reiterated in Figure 5 legend. Since we did not observe differences between sexes, the data reflects both sexes combined.
Field-collected samples	The study did not involve field samples
Ethics oversight	All animal experiments were conducted in compliance with the University of California San Diego Institutional Care and Use Committee (IACUC) and all experimental procedures were approved by the UC San Diego IACUC.

Note that full information on the approval of the study protocol must also be provided in the manuscript.
